# Supplementary material for: Induction of Stem-Cell-Derived Cardiomyogenesis by Fibroblast Growth Factor 10 (FGF10) and Its Interplay with Cardiotrophin-1 (CT-1)
Source: Biology (Basel). 2022 Mar 30;11(4):534. doi: 10.3390/biology11040534 (PMC9026462; doi:10.3390/biology11040534)

Figure S1. Full western blot images used in Figures 3 and 5 as representative (N1) and other replicates (N2, N3 and N4) quantified for their respective graphs. Cells dissociated from embryoid bodies were treated from day 9 to day 12 with FGF10 and/or CT-1 as well as vehicle. Protein was extracted on day 12 and subjected to Western blot to analyze the status of α-actinin, Aurora B, MLC-2v, Troponin T2, NKX2-5, YAP-1, MLC-2a, and GATA4 together with their corresponding housekeeping proteins (vinculin and β-actin).

α-actinin and housekeeping control WB replicates

α-actinin (104 kDa) N1, representative

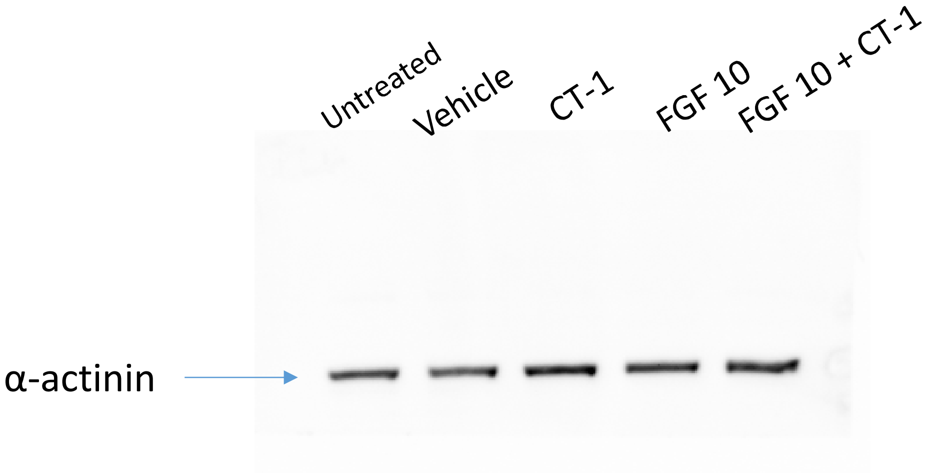

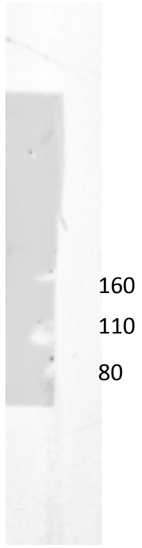

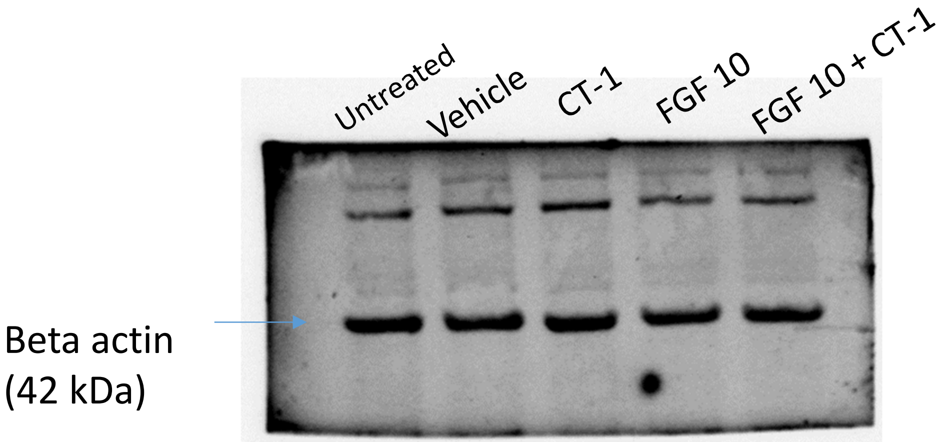

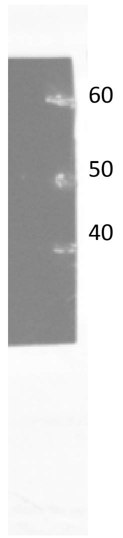

as housekeeping of α-actinin (N1,representative)

α-actinin (104 kDa), N2

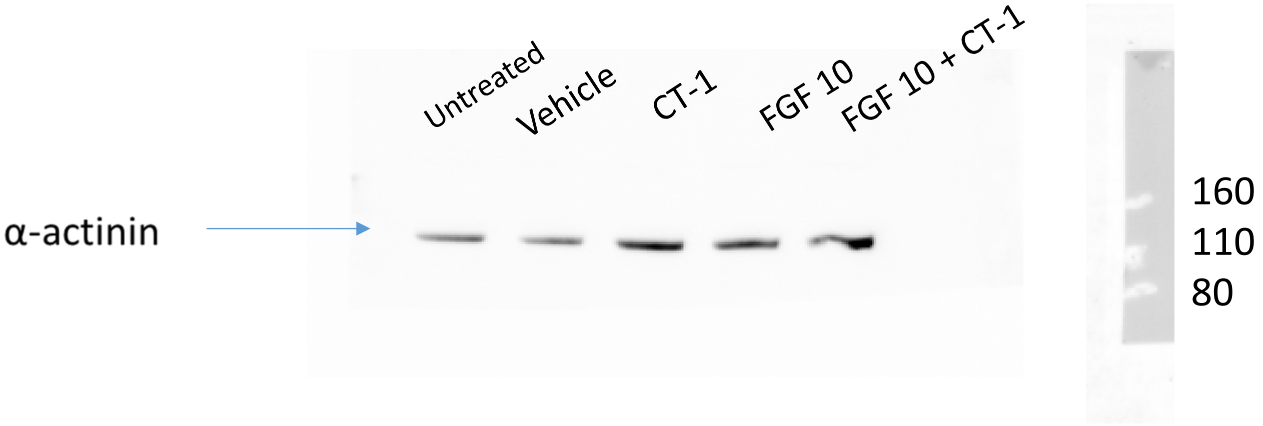

Beta actin
(42 kDa)
as housekeeping of α-actinin (N2)

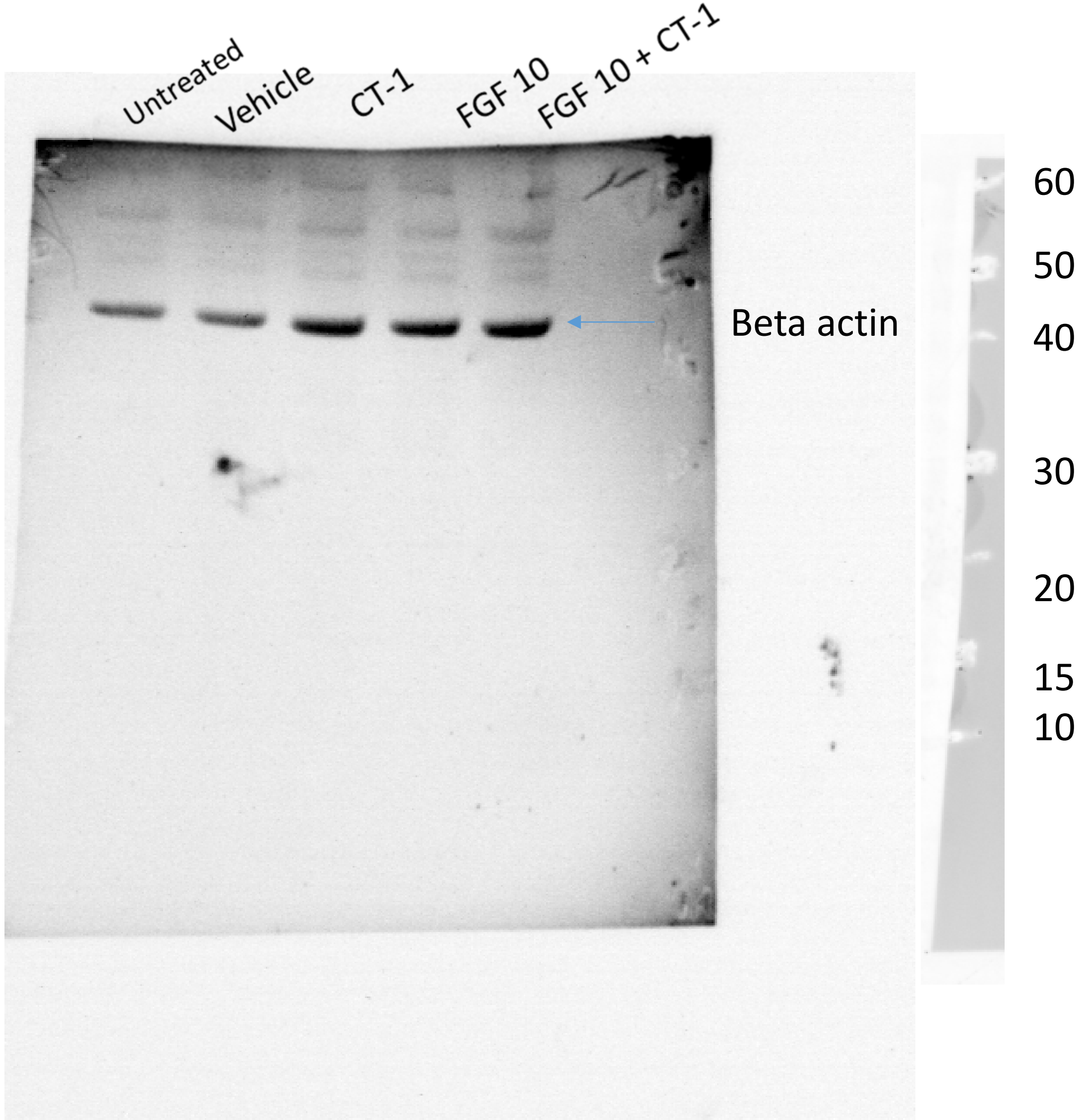

α-actinin (104 kDa), N3

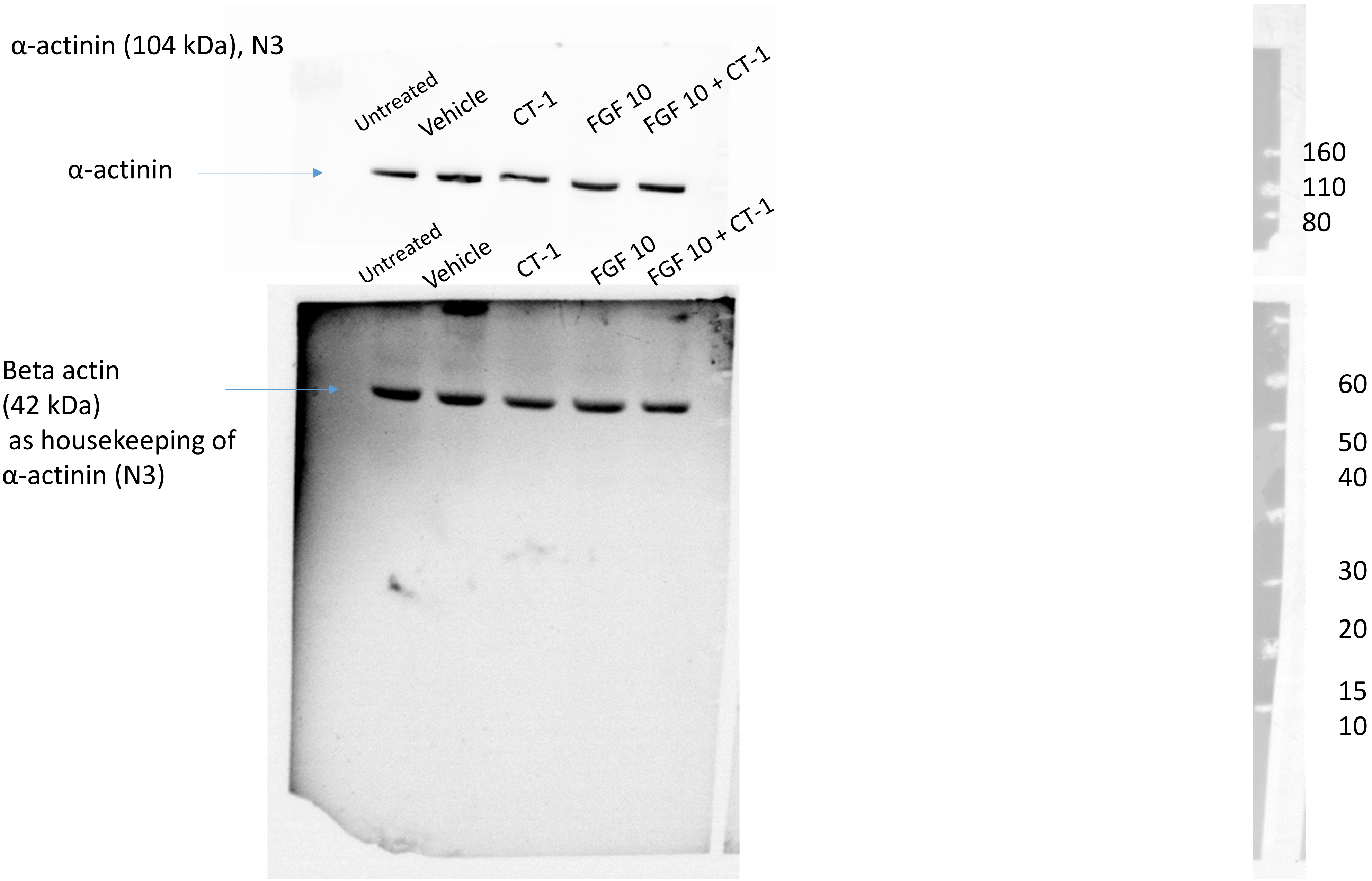

α-actinin (104 kDa), N4

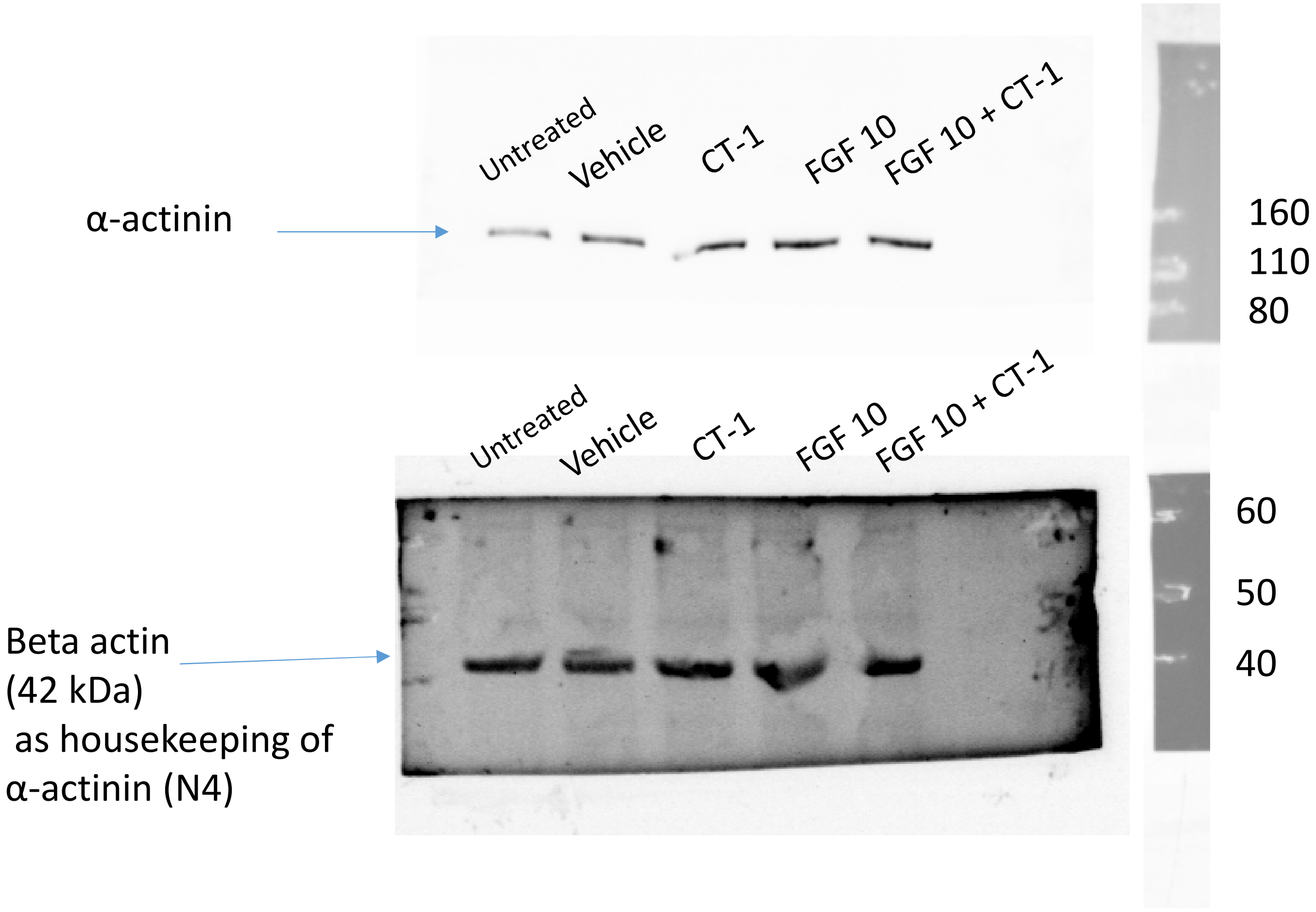

Aurora-B and housekeeping control WB replicates

Aurora B (39 kDa), N1, representative

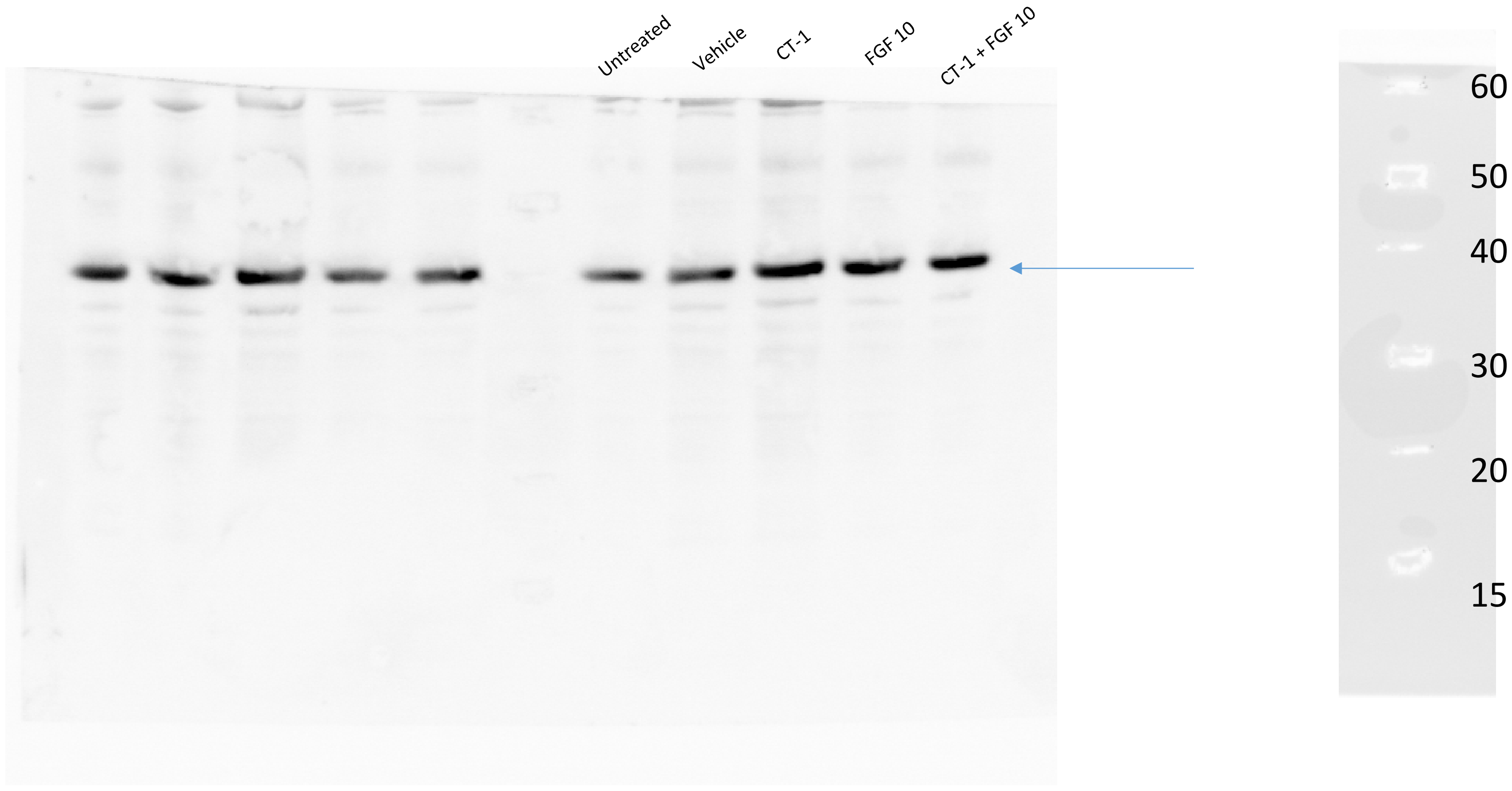

Vinculin 124 kDa as housekeeping of Aurora B (N1,representative)

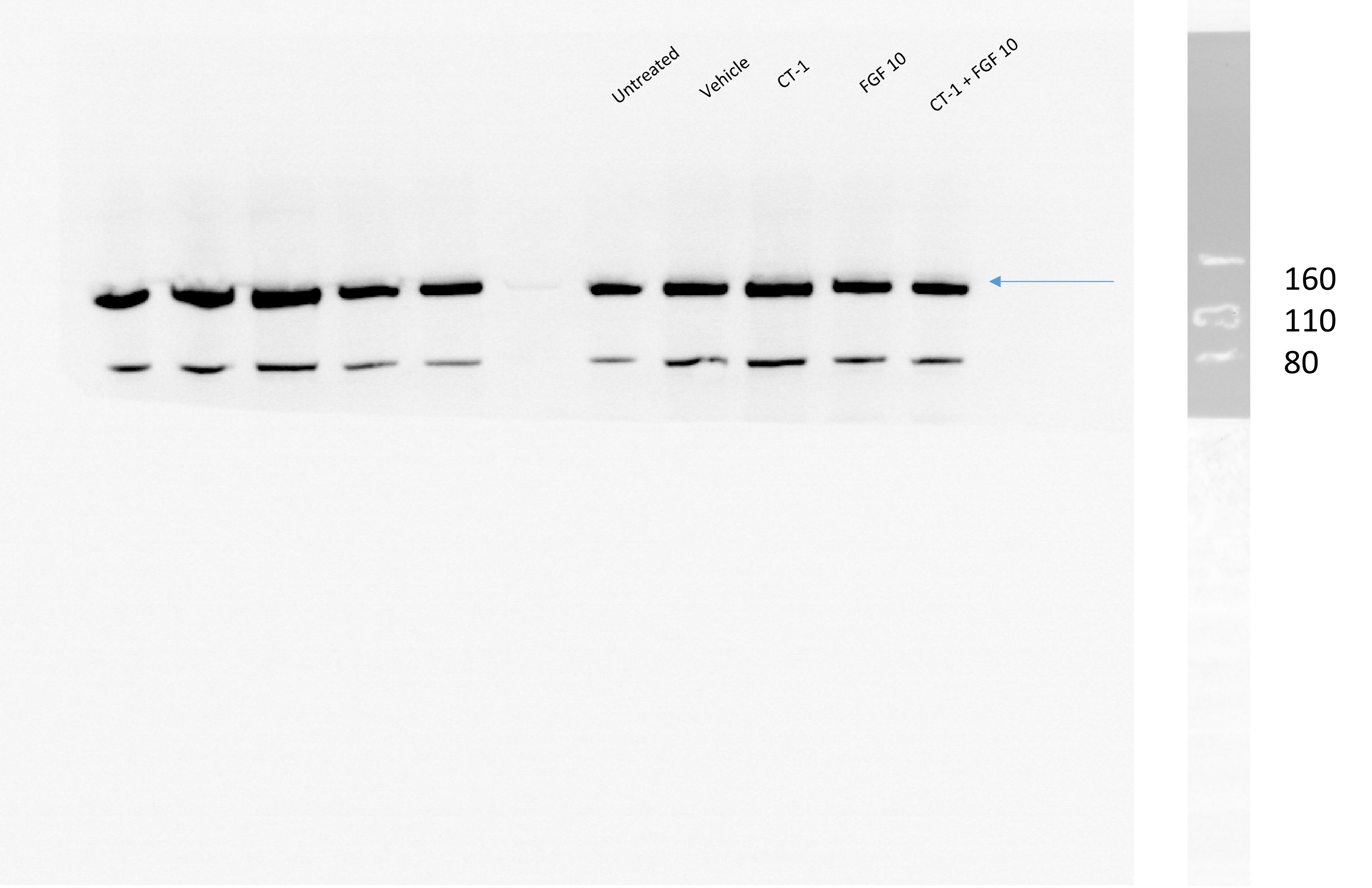

Aurora B (39 kDa), N2

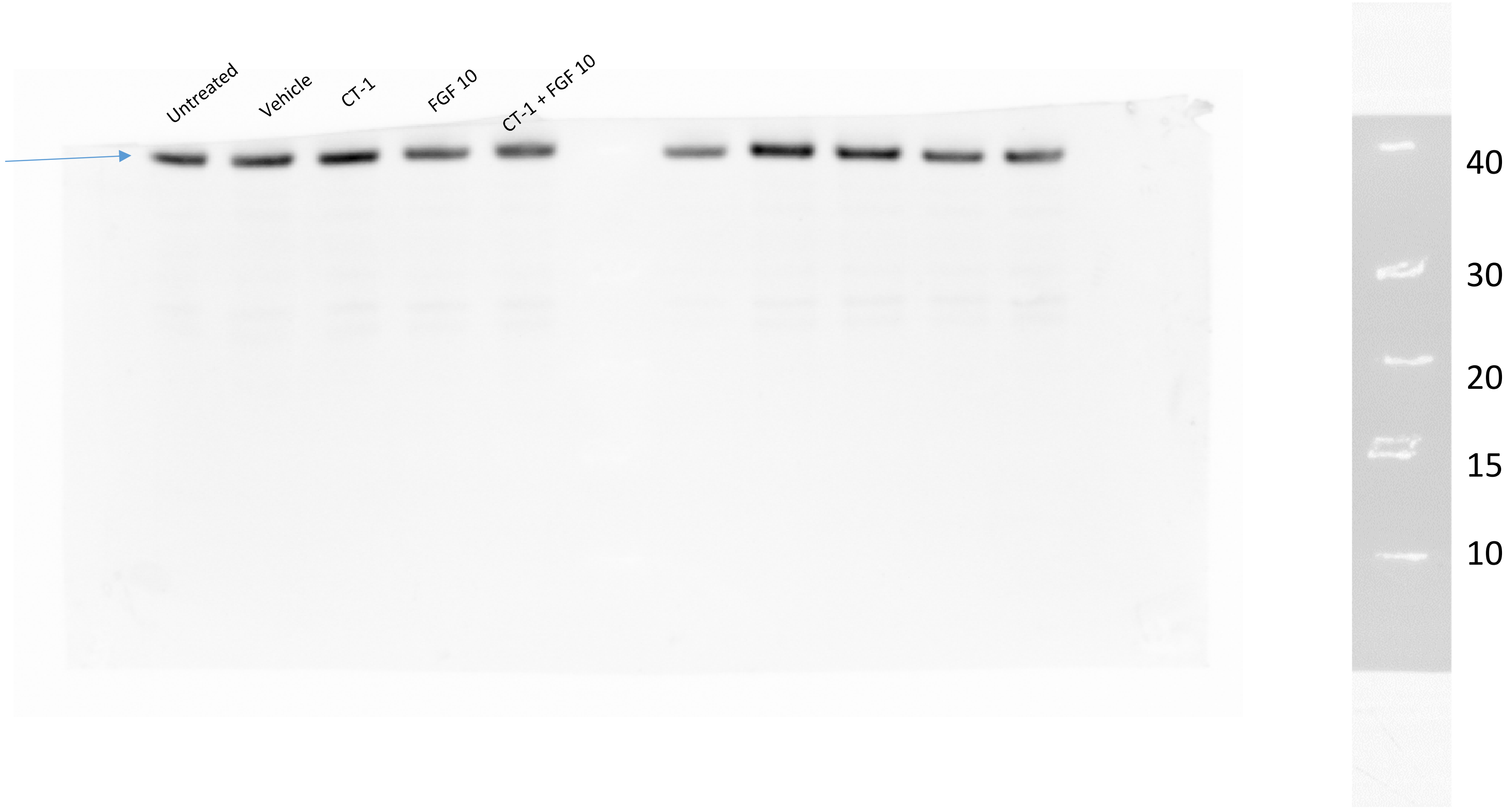

Vinculin for Aurora B, N2

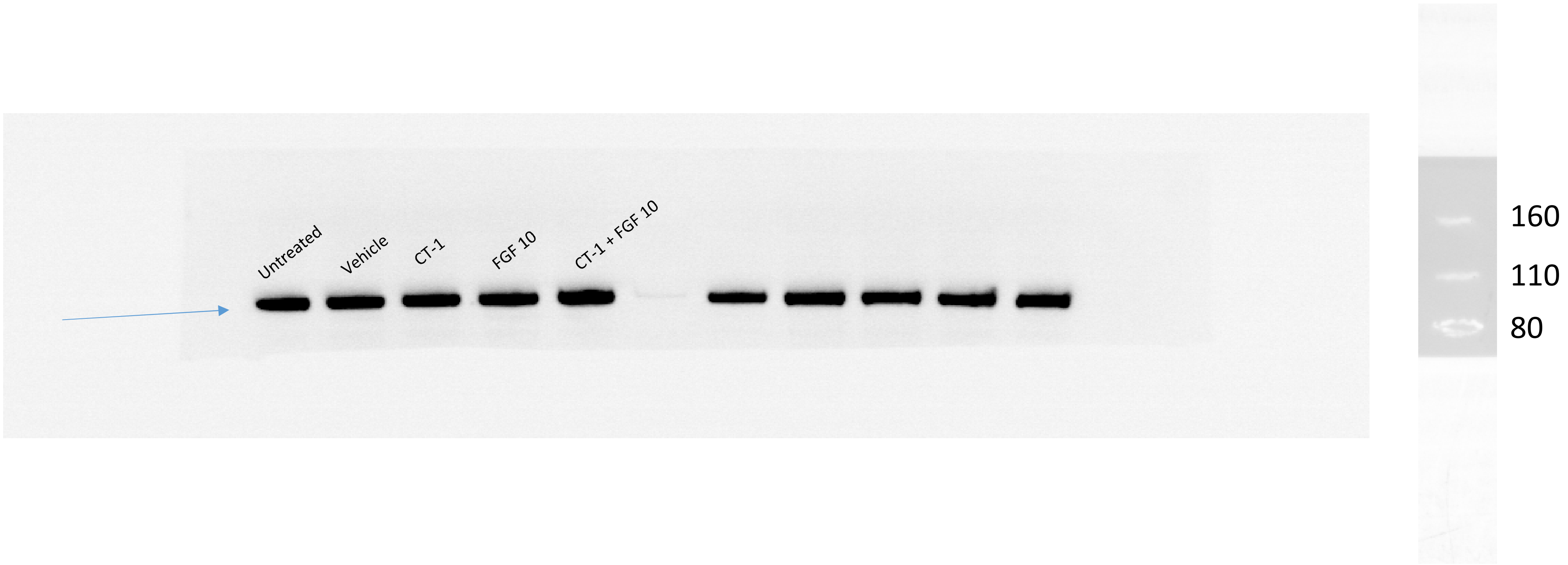

Aurora B (39 kDa), N3

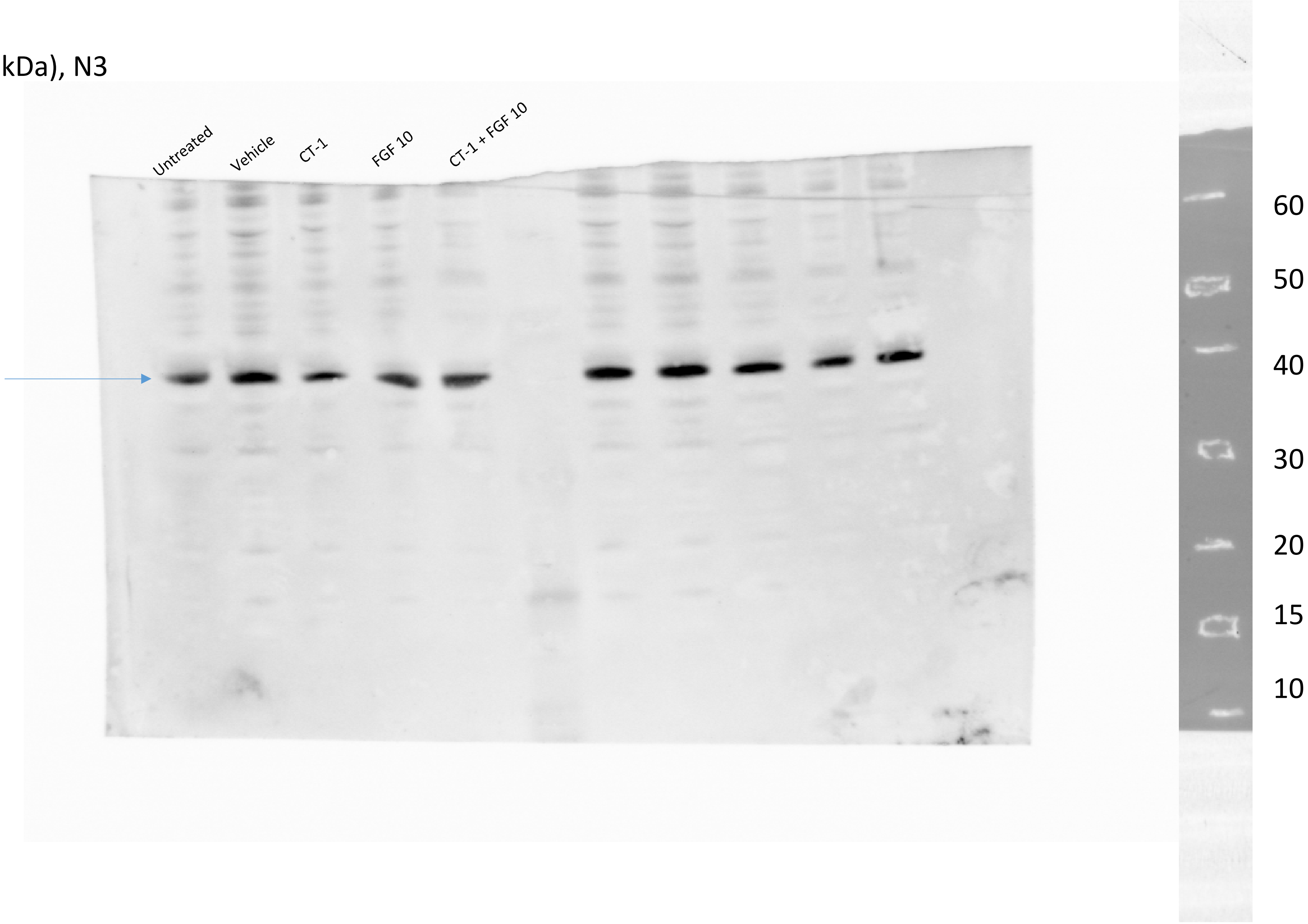

Vinculin for Aurora B, N3

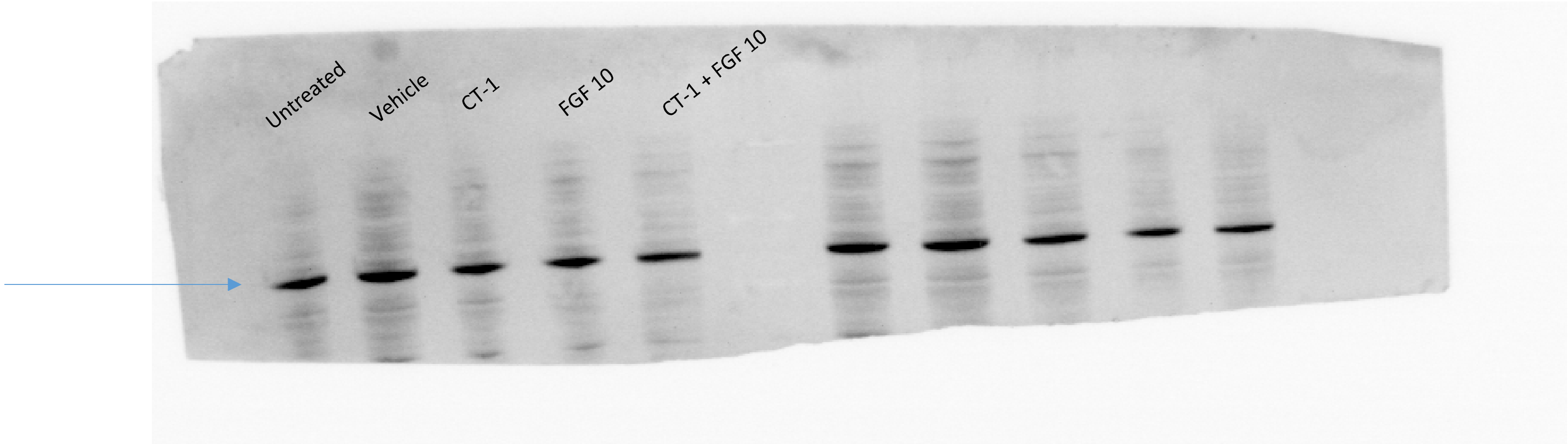

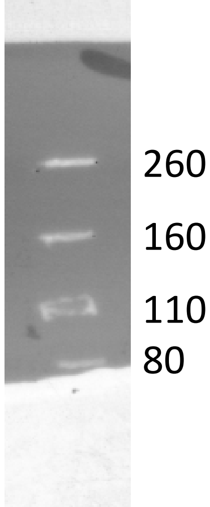

Aurora B (39 kDa), N4

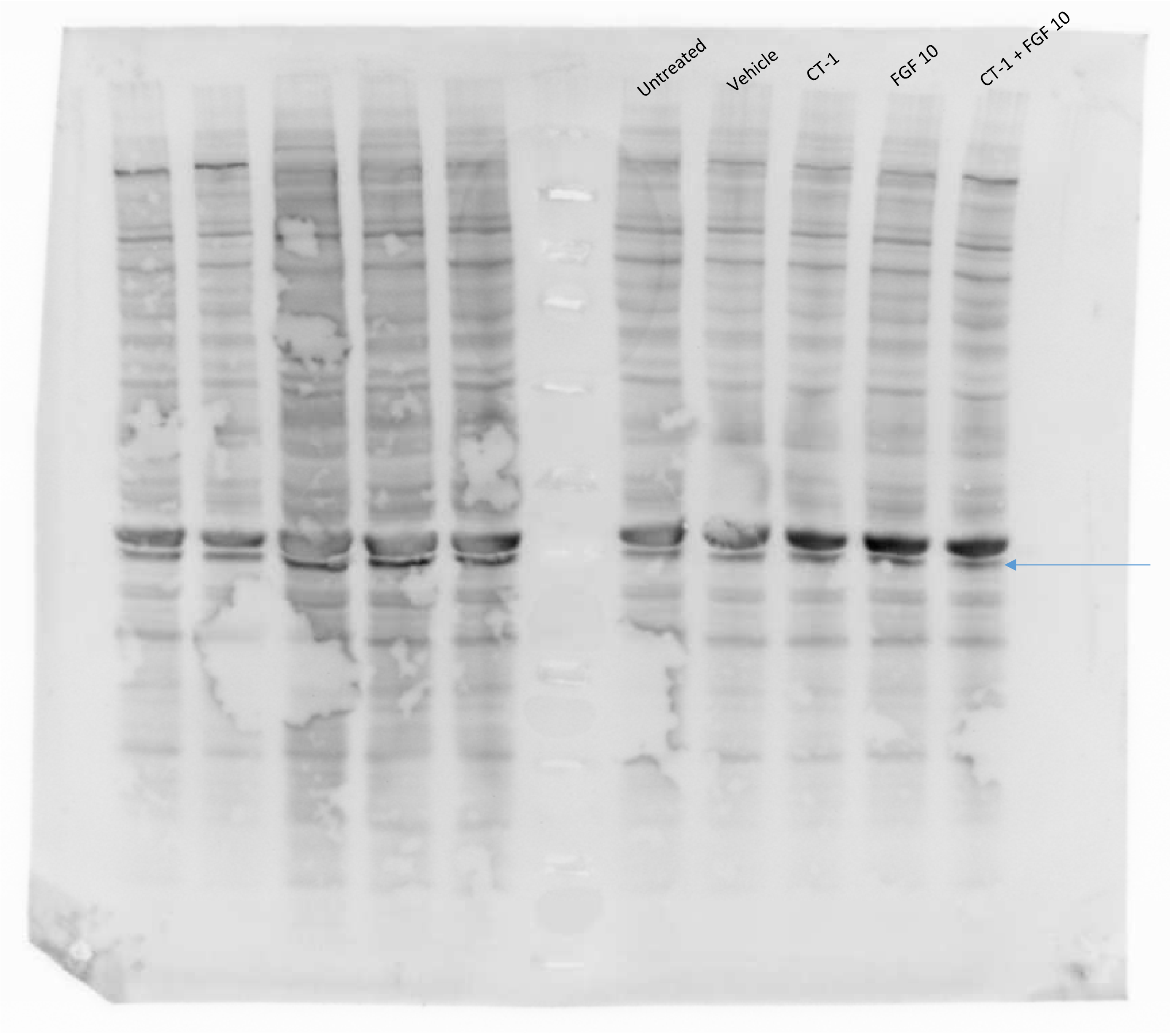

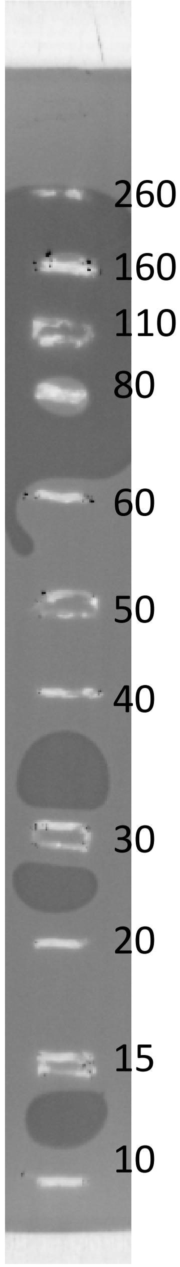

Vinculin for Aurora B, N4

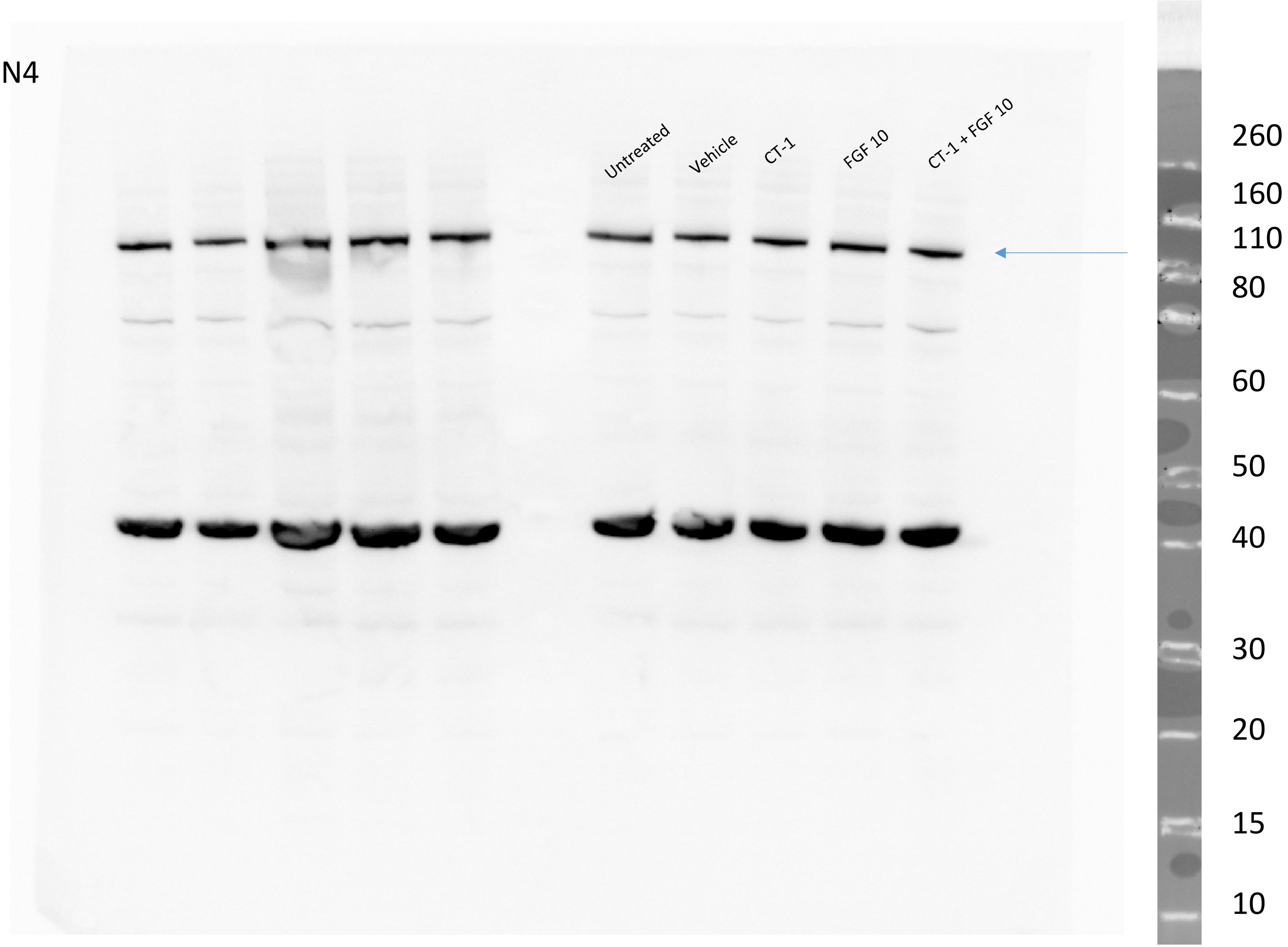

MLC2v and Housekeeping control WB replicates

MLC2v (19 kDa), N1, representative

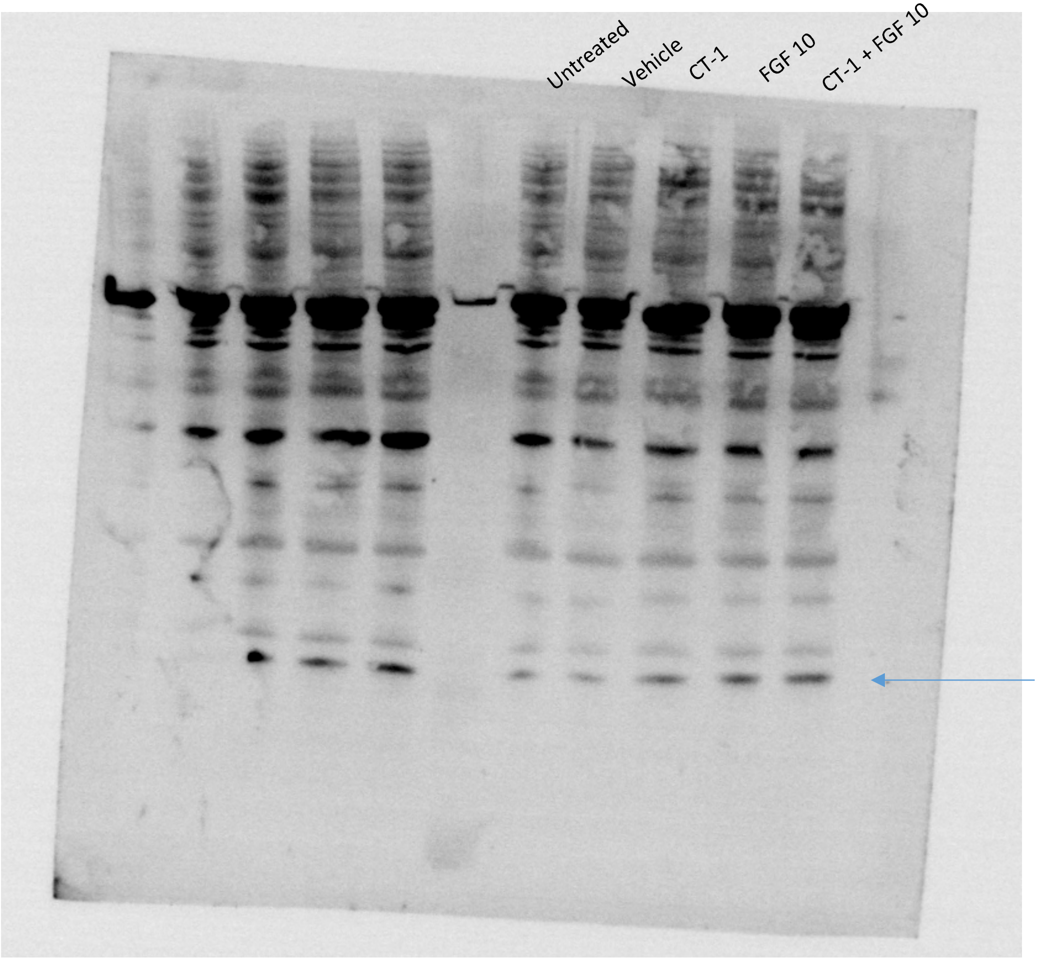

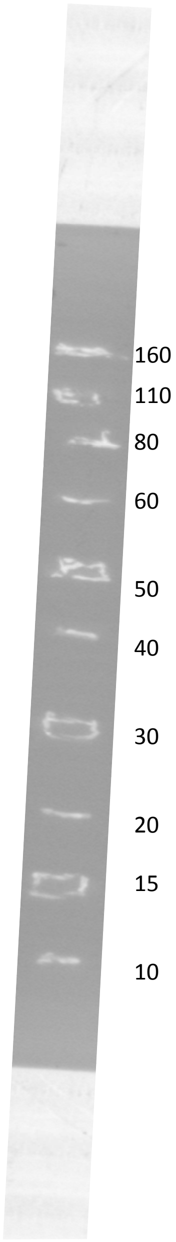

Vinculin (124kd)
Housekeeping of MLC2v (N1, representative)

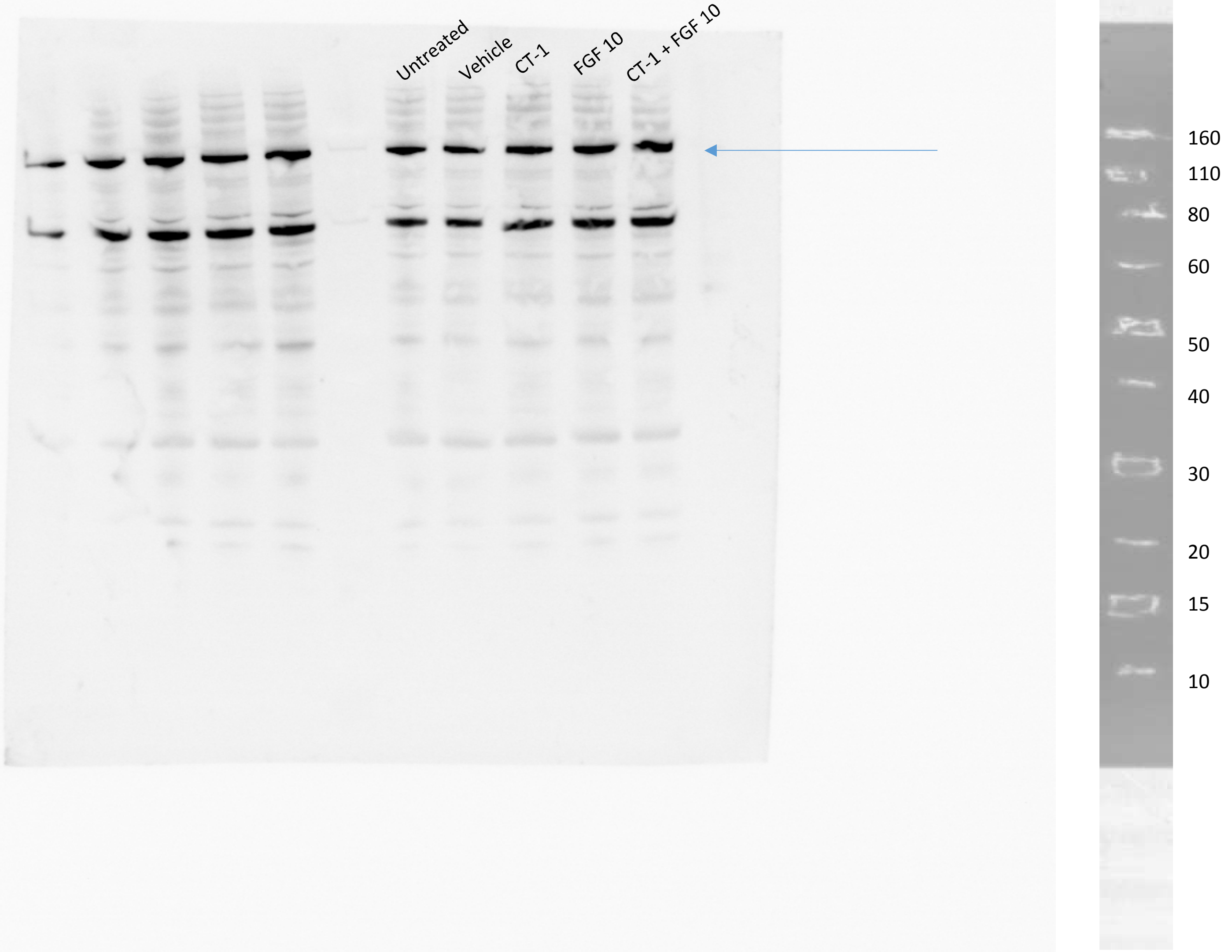

MLC2v, N2

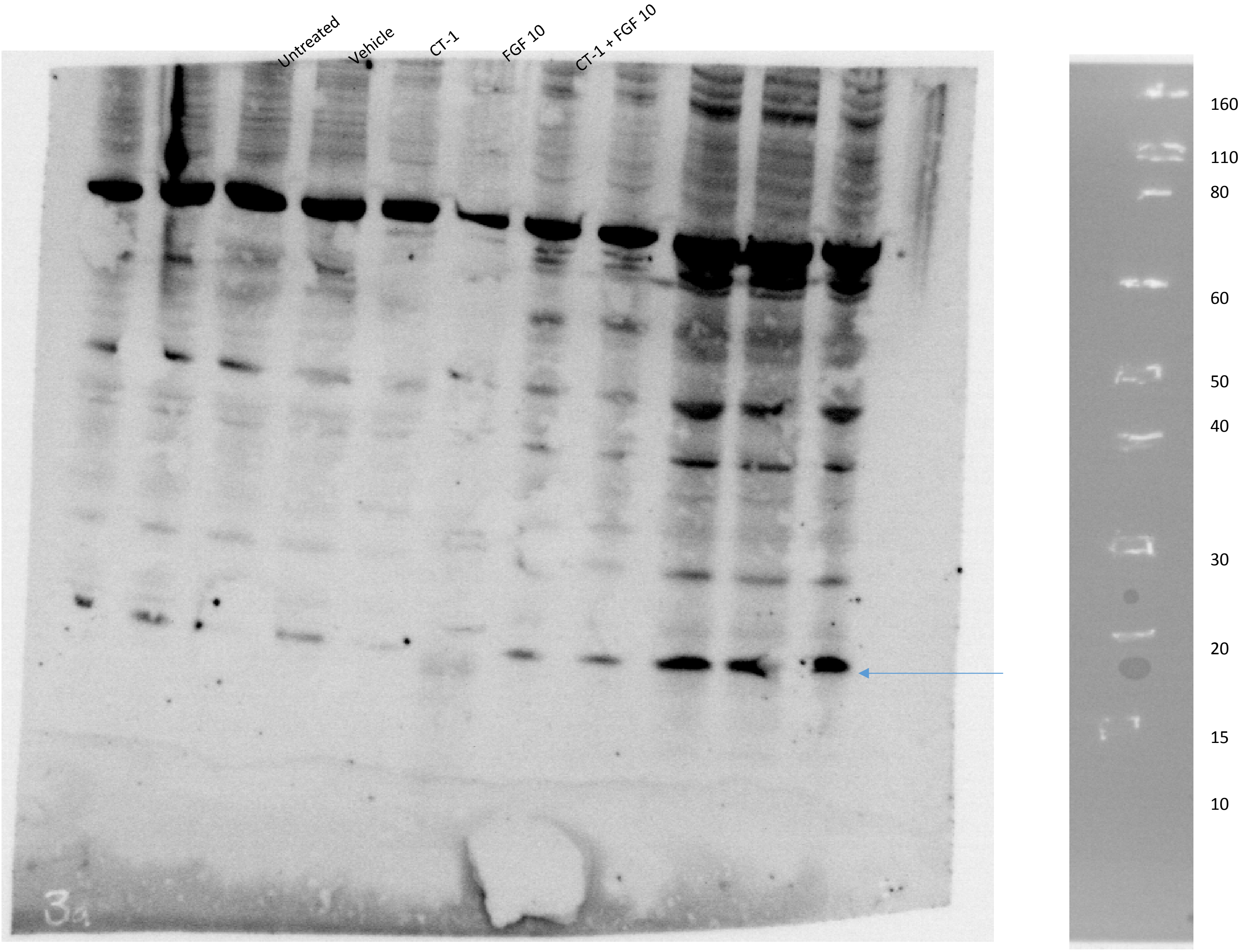
Untreated
Vehicle
CT-1
FGF 10
CT-1 + FGF 10
160
110
80
60
50
40
30
20
15
10

Vinculin (124kd)
Housekeeping of MLC2v, N2

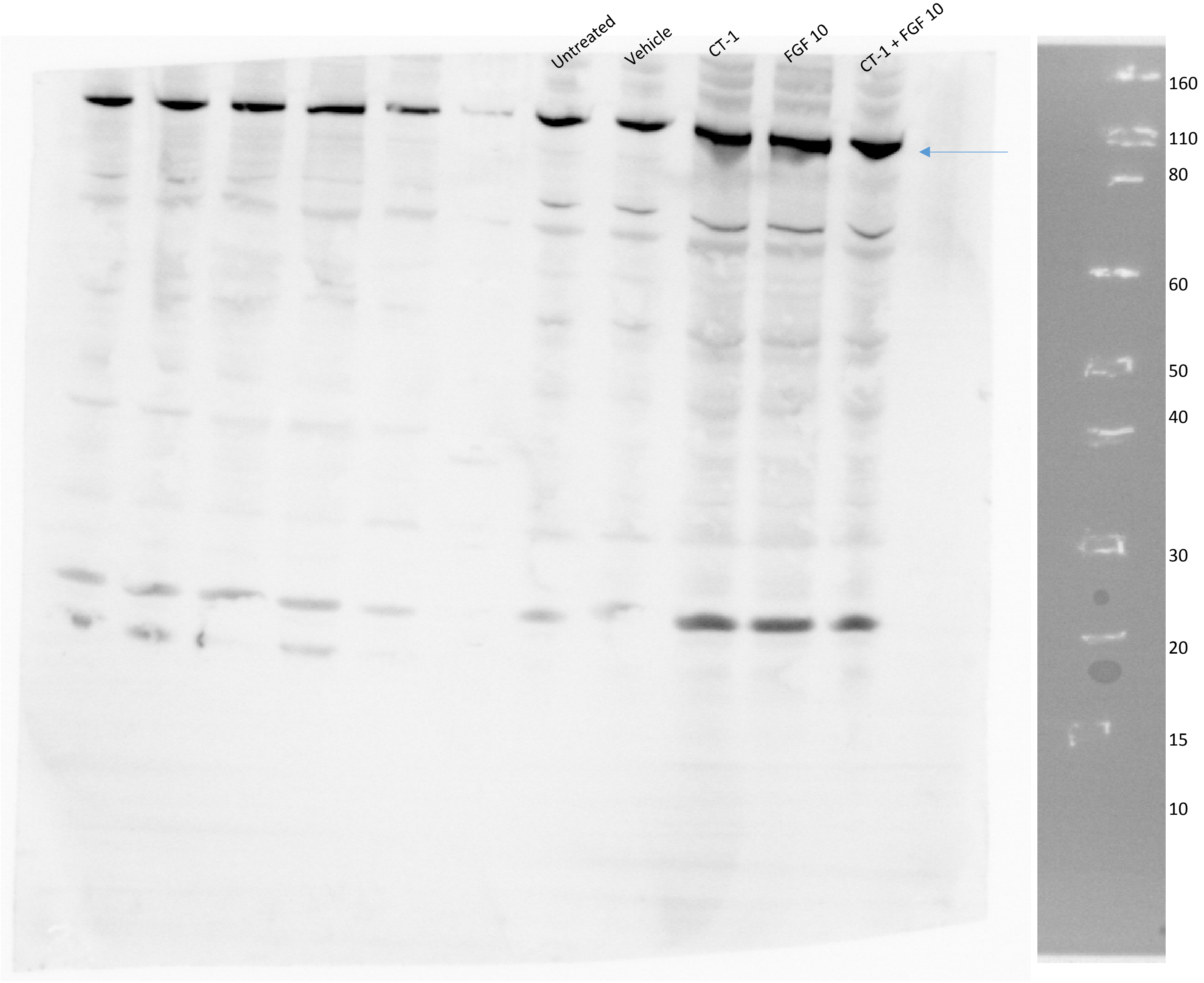

MLC2v, N3

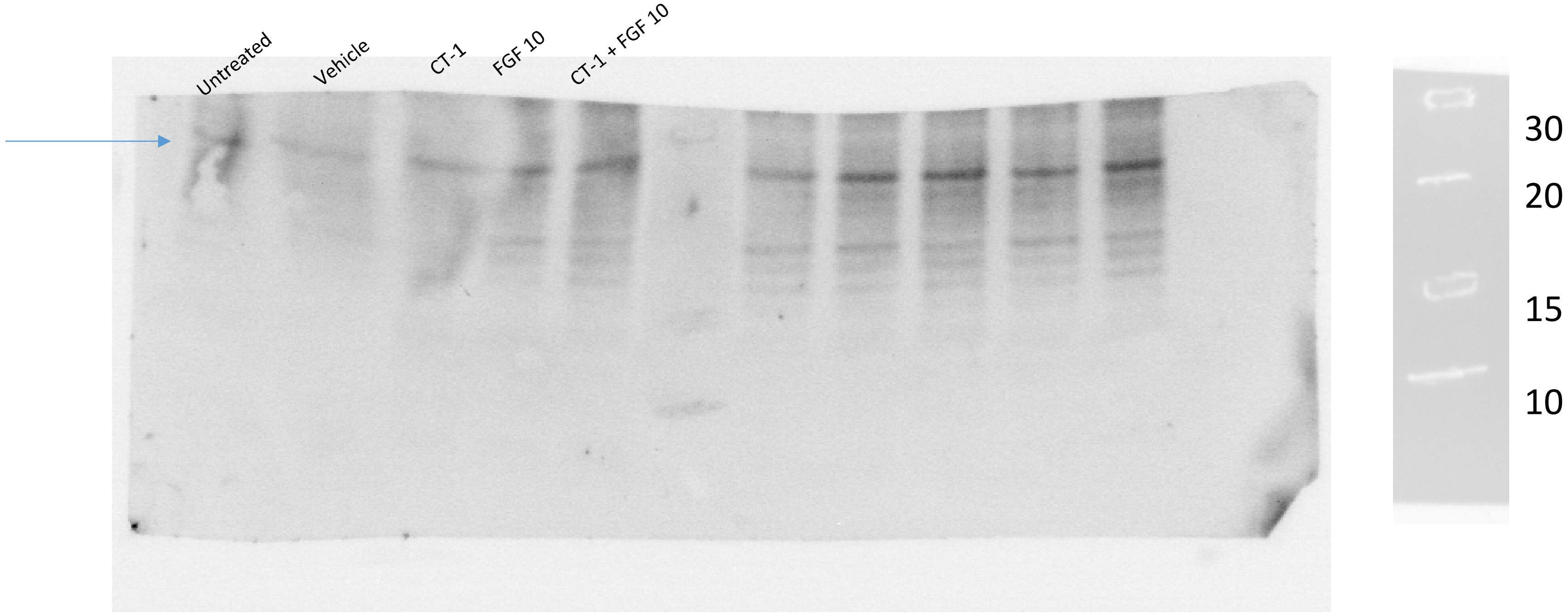

Vinculin Housekeeping of MLC2v N3

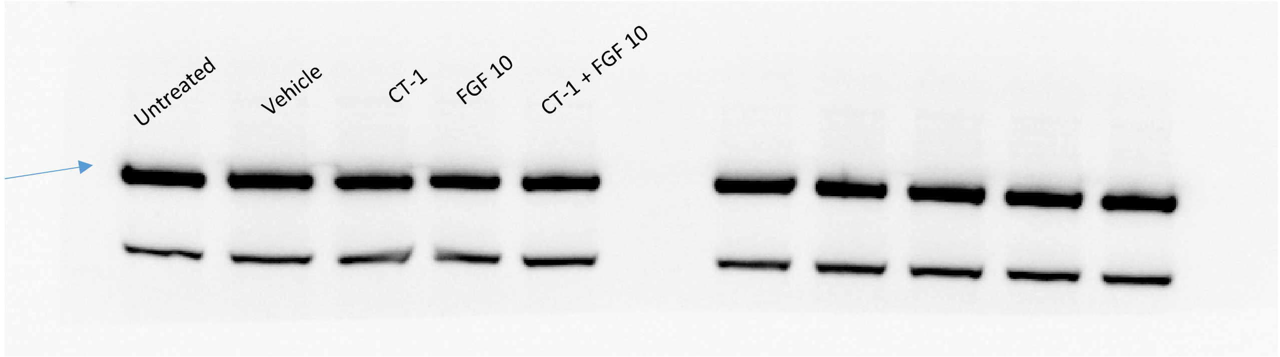

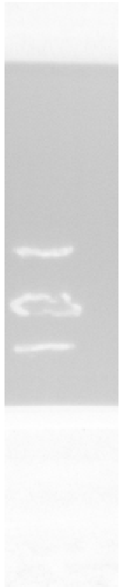

160
110
80

MLC2v, N4

MYL2 (MLC2v)

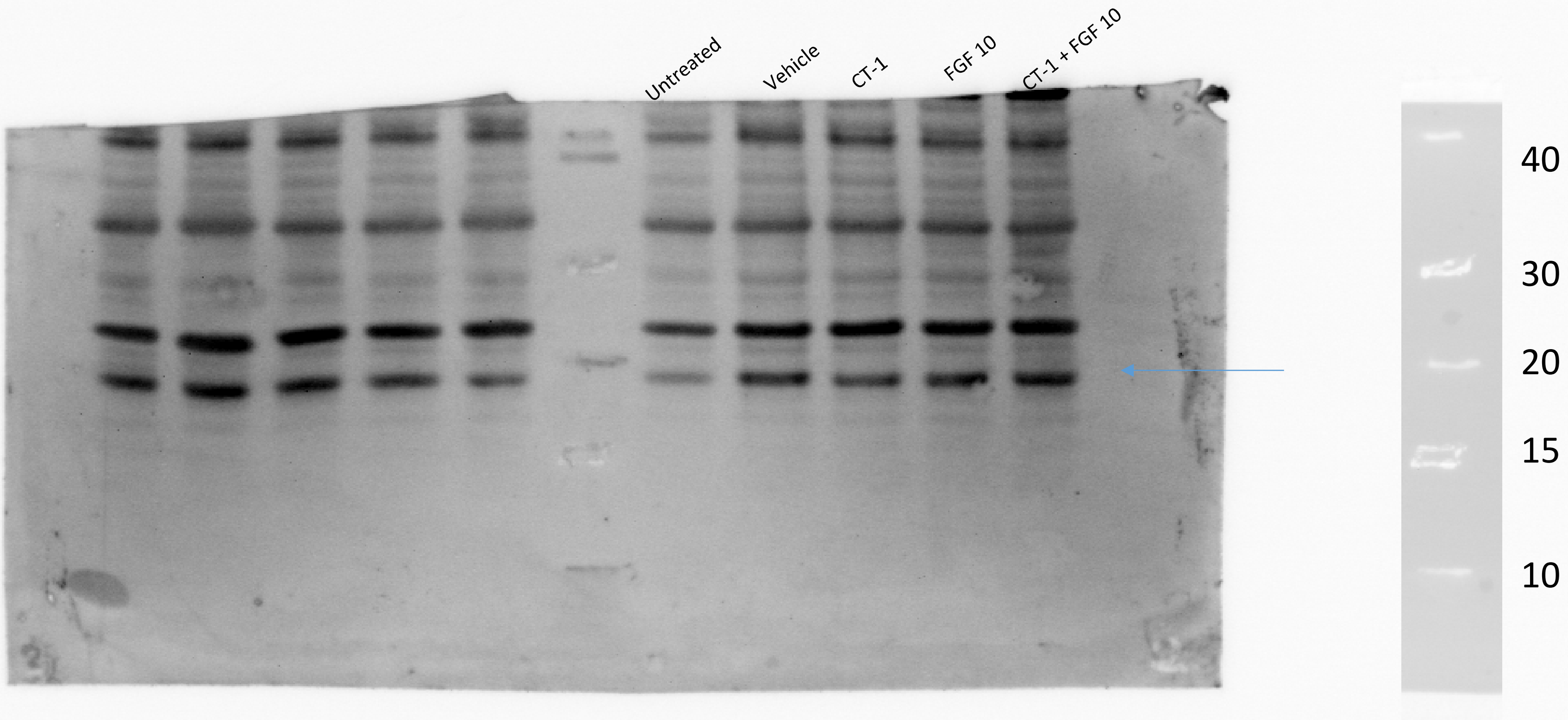

Vinculin Housekeeping of MLC2v N4

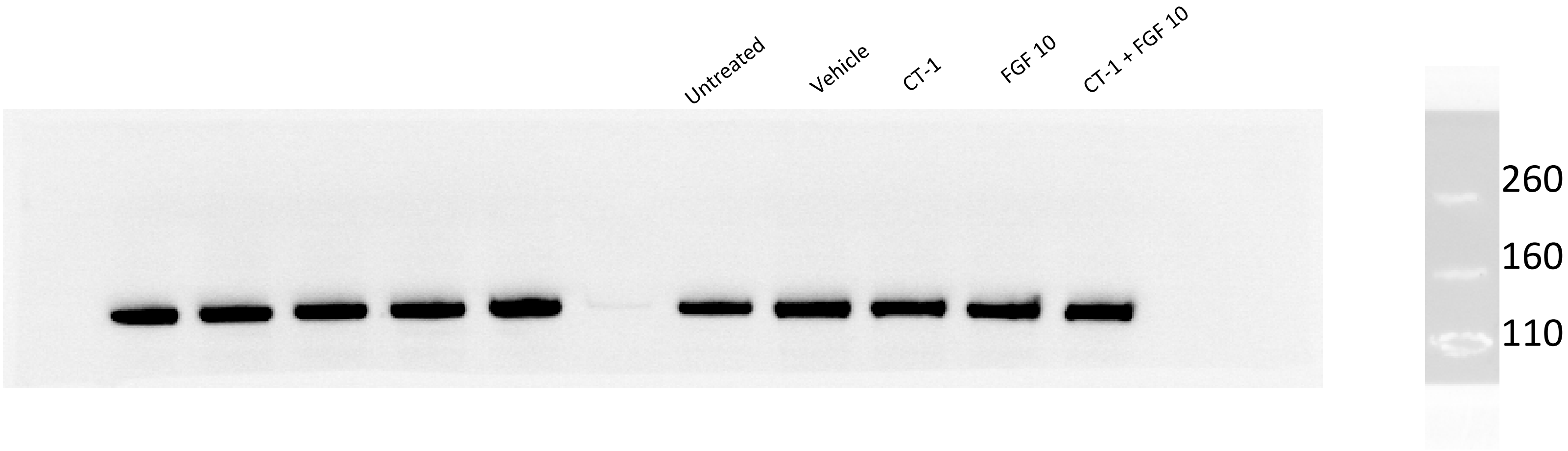

Troponin T and housekeeping control WB replicates

Troponin T (36 kDa)
Representative, N1

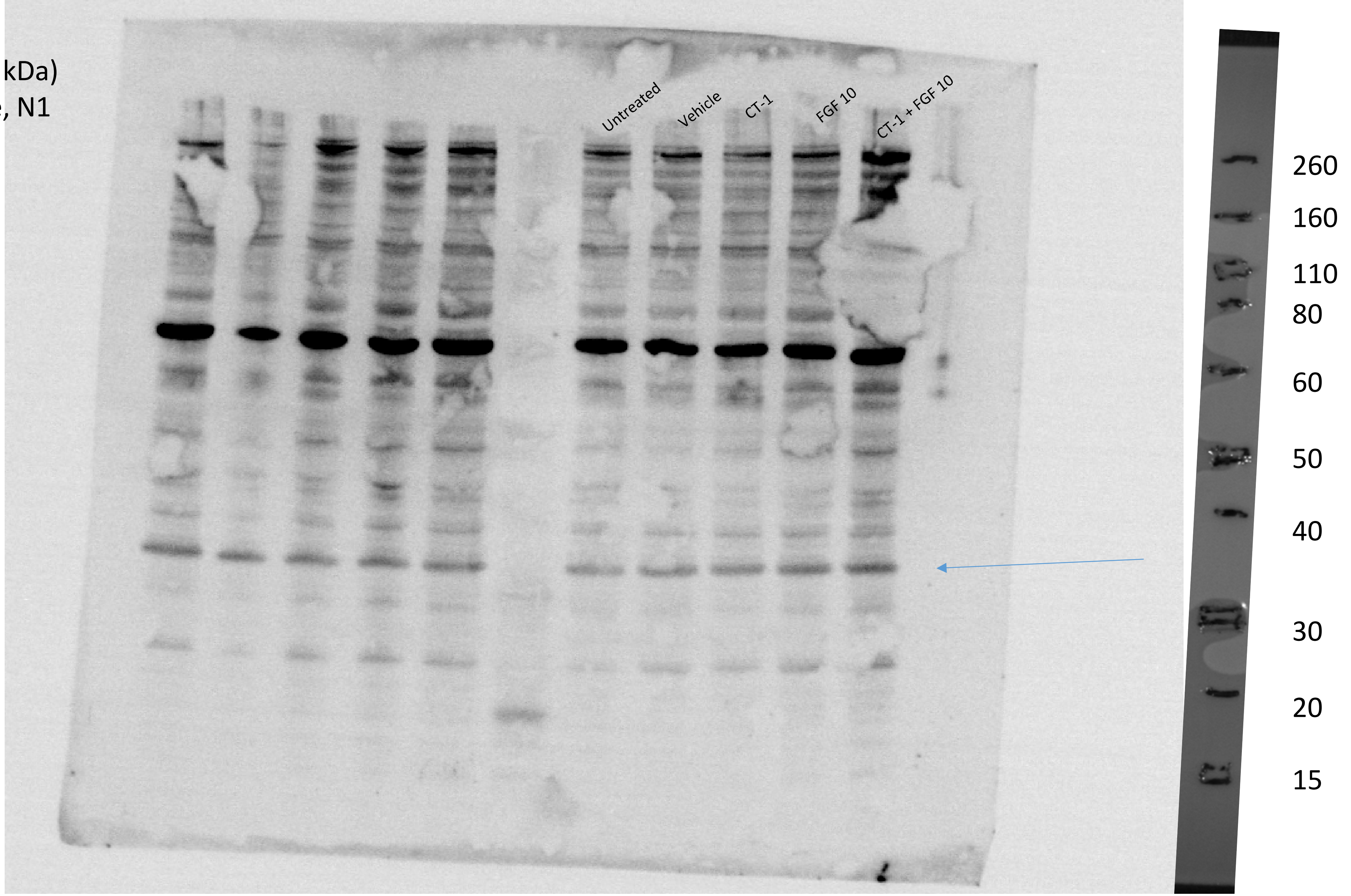

Beta actin (42 kDa)
Housekeeping of Troponin T2 (N1, representative)

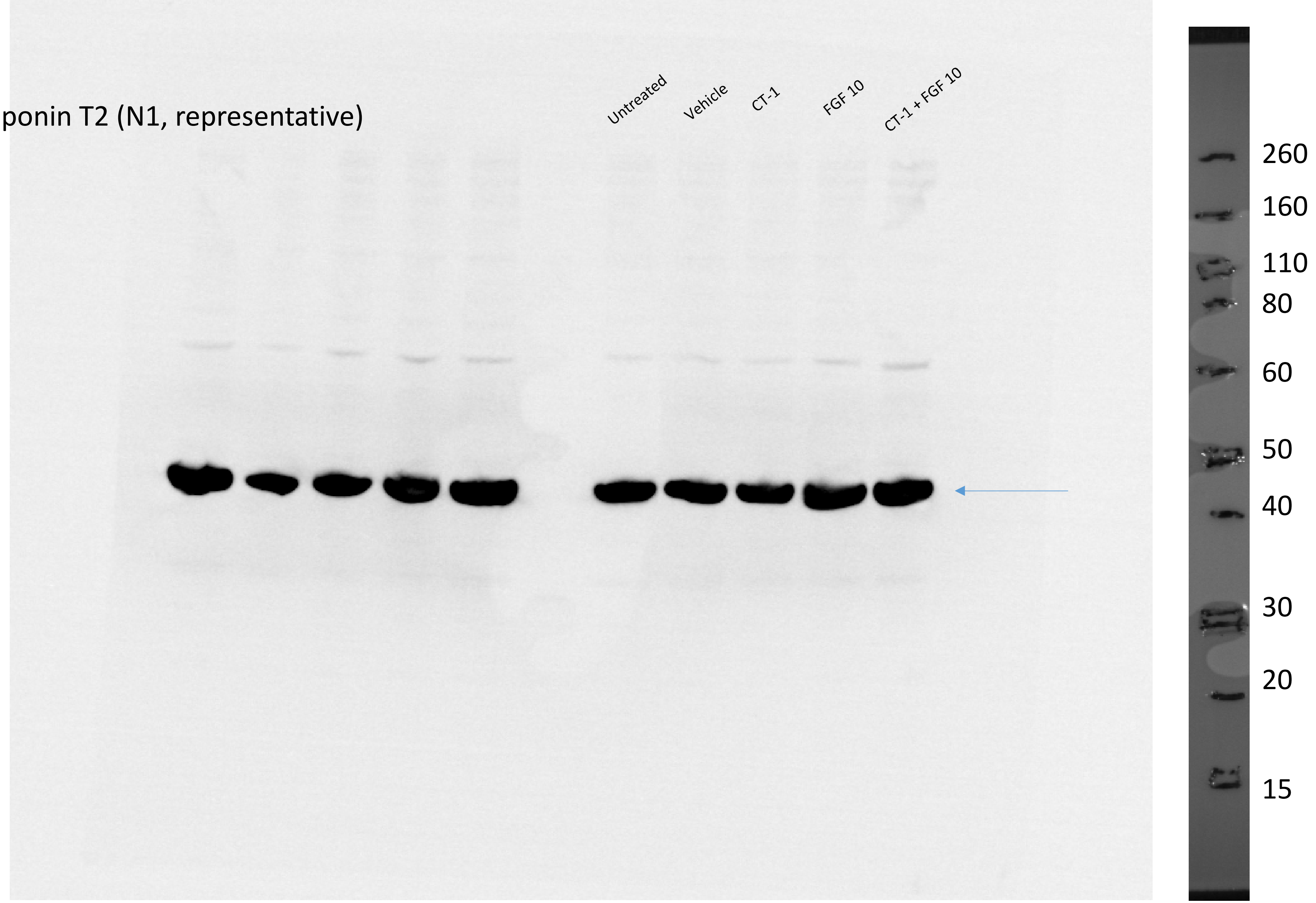

Troponin T, N2 and N3

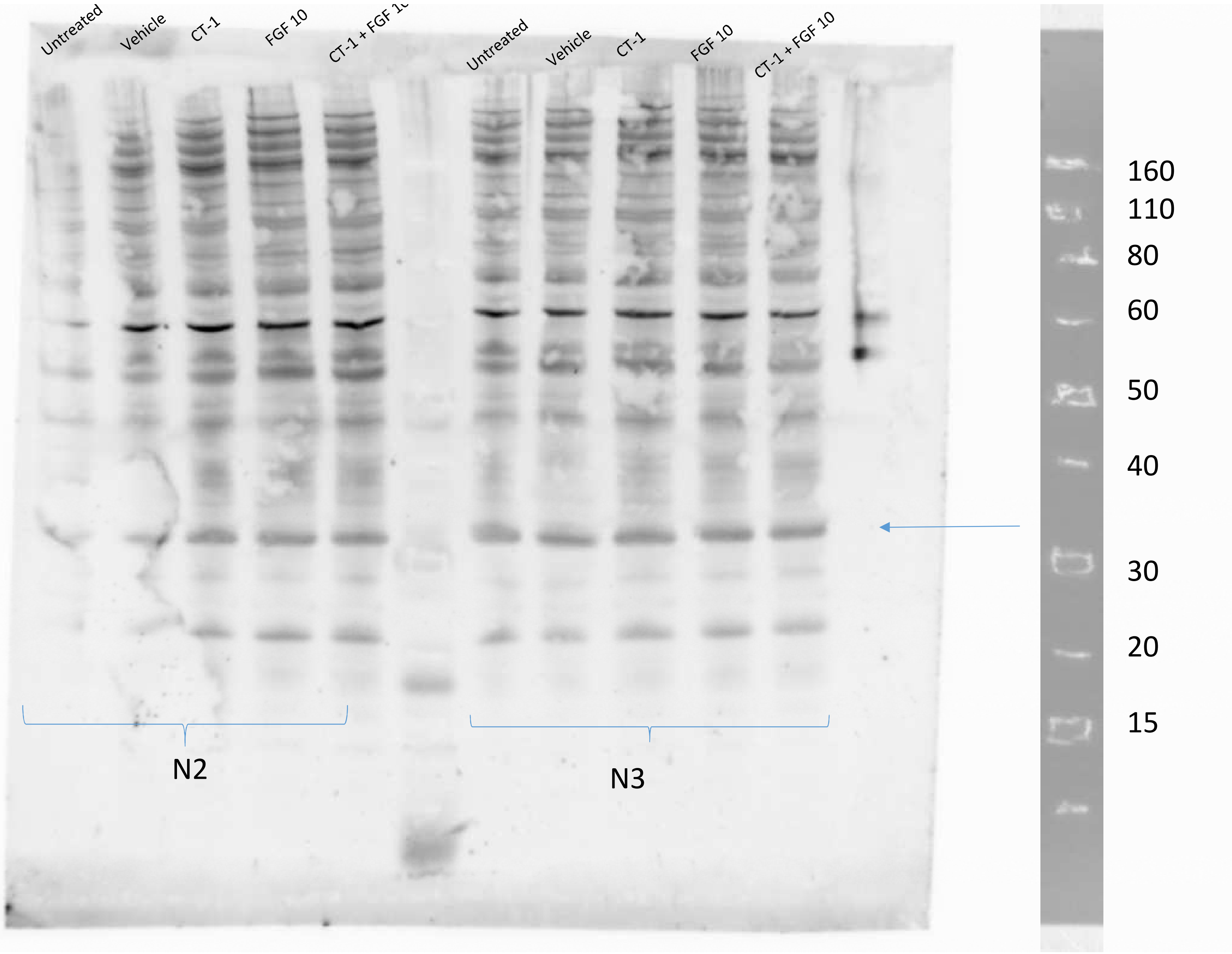

Beta actin (42 kDa)
Housekeeping of Tropo
(N2 and N3)

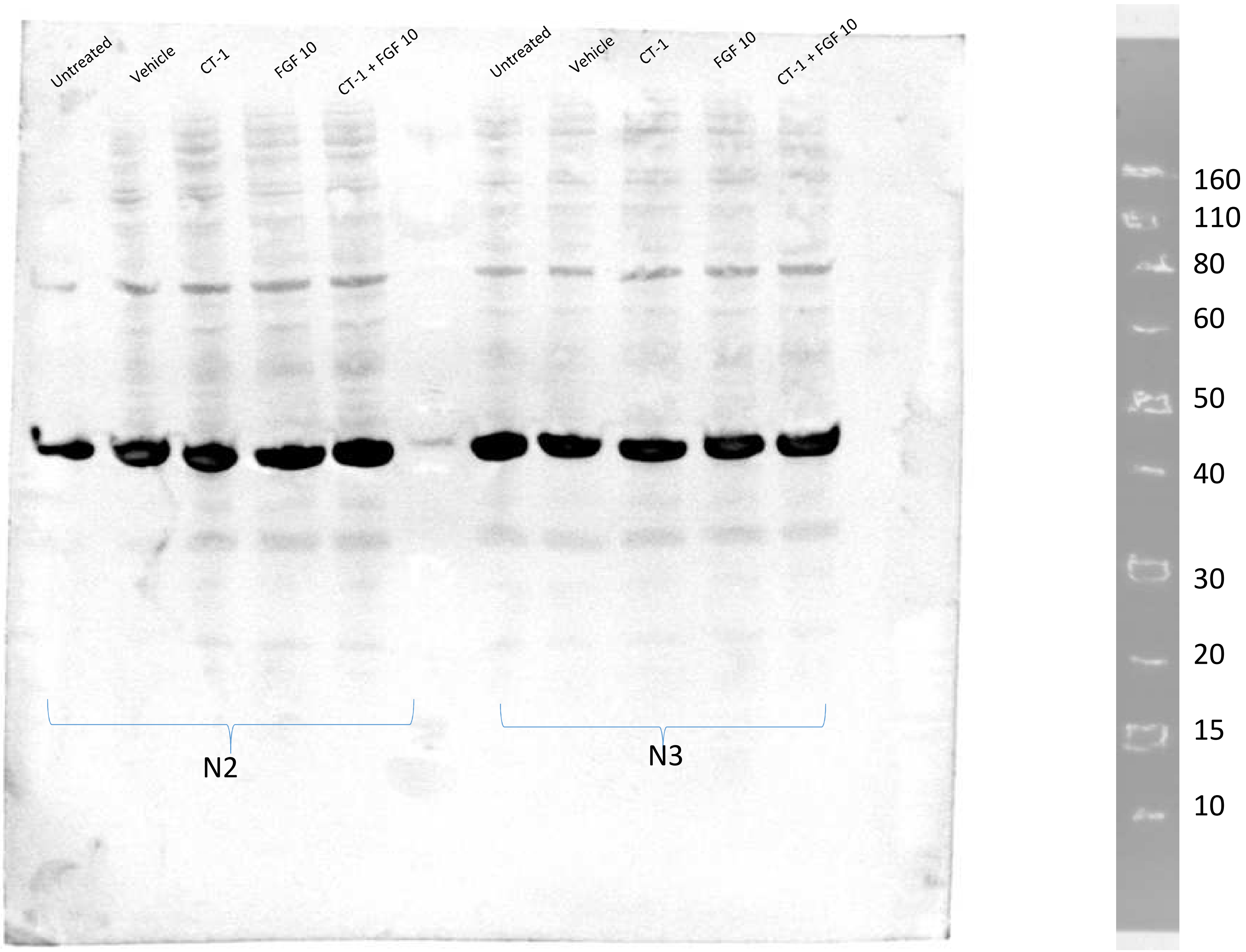

Troponin T, N4

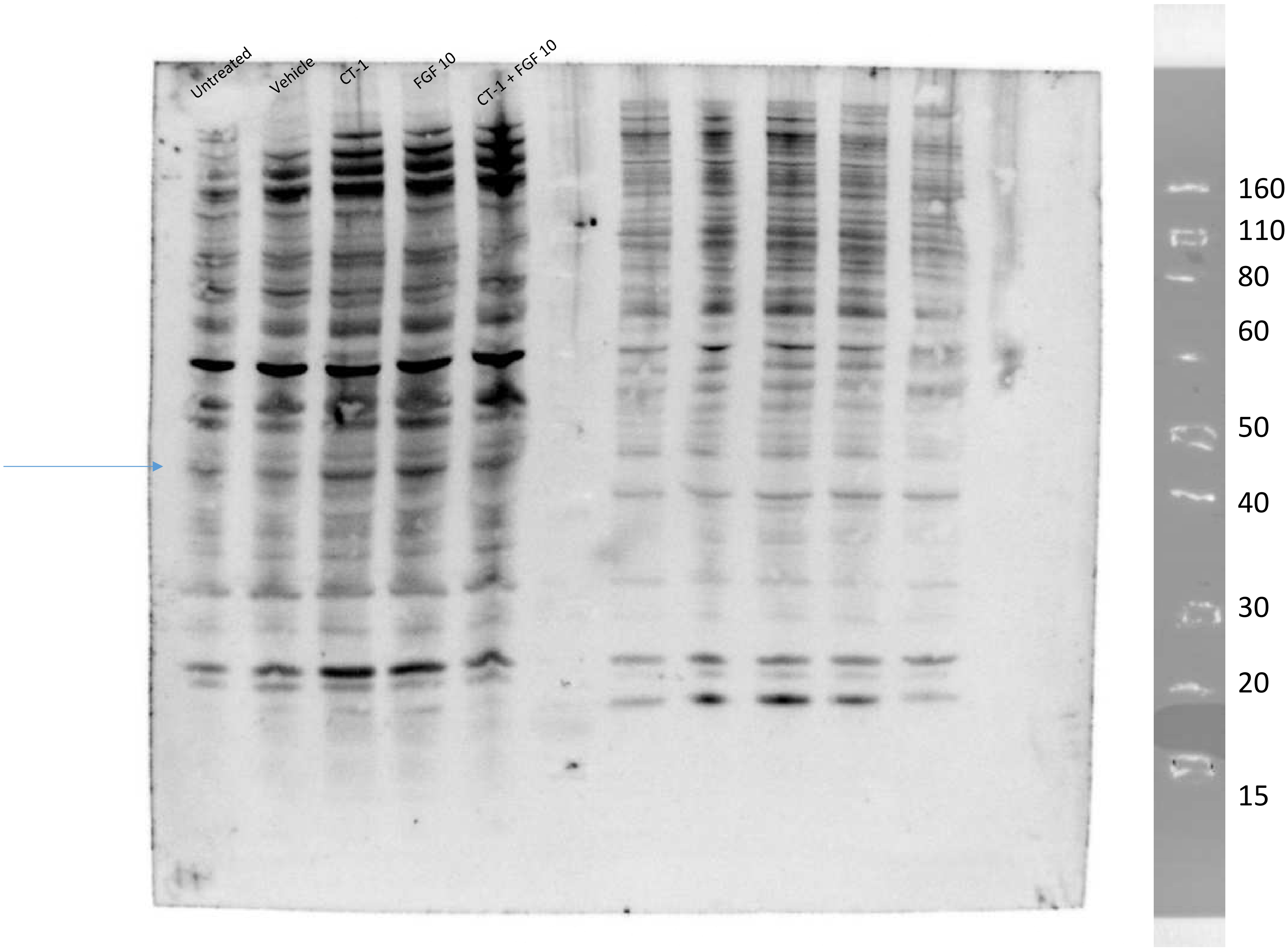

Beta actin (42 kDa)Housekeeping of Troponin T2 (N4)

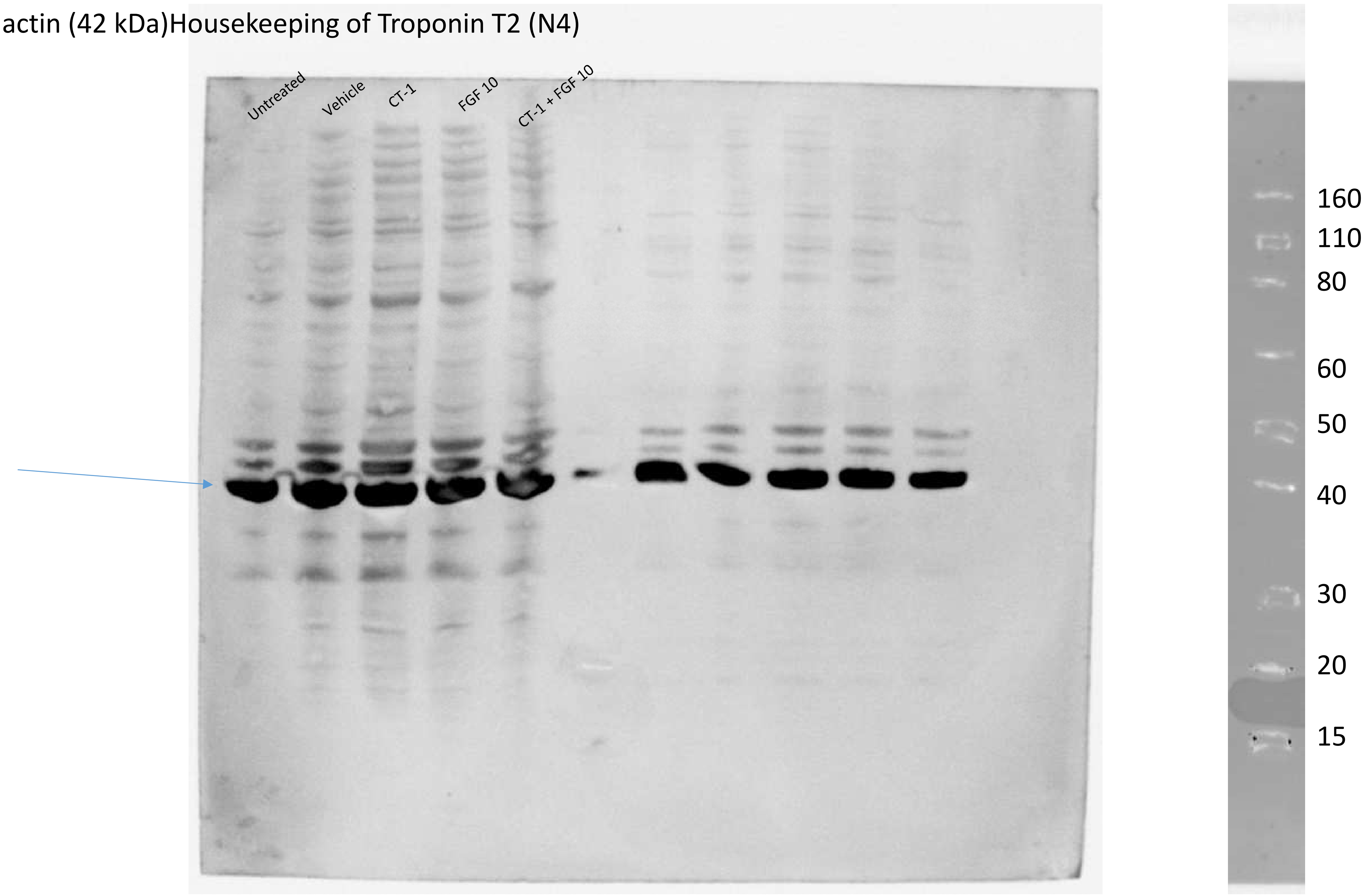

NKX2-5 and housekeeping control WB replicates

NKX2-5 (34kDa)
N1, representative

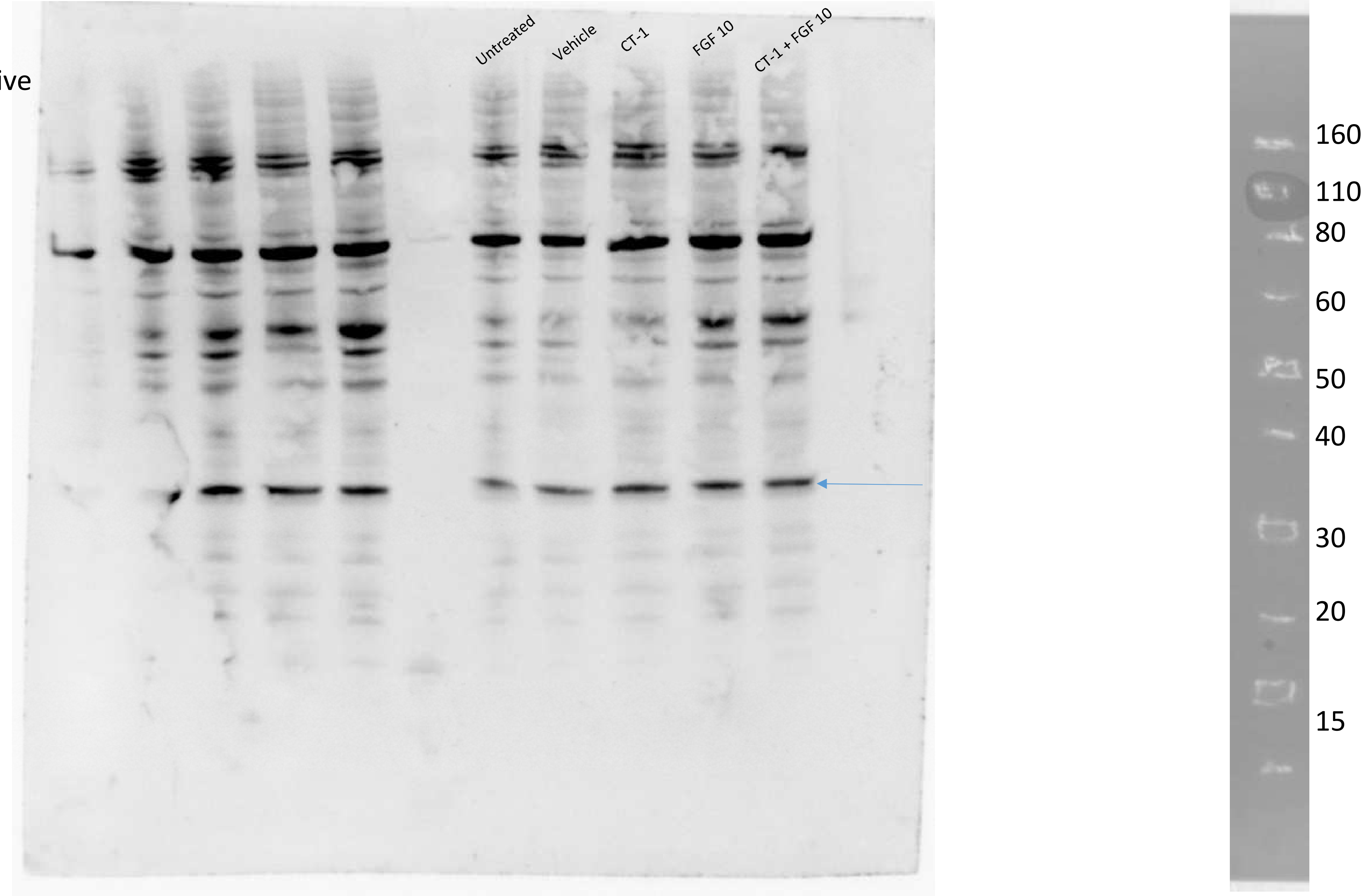

Beta actin (42 kDa)
Housekeeping of NKX2-5
N1

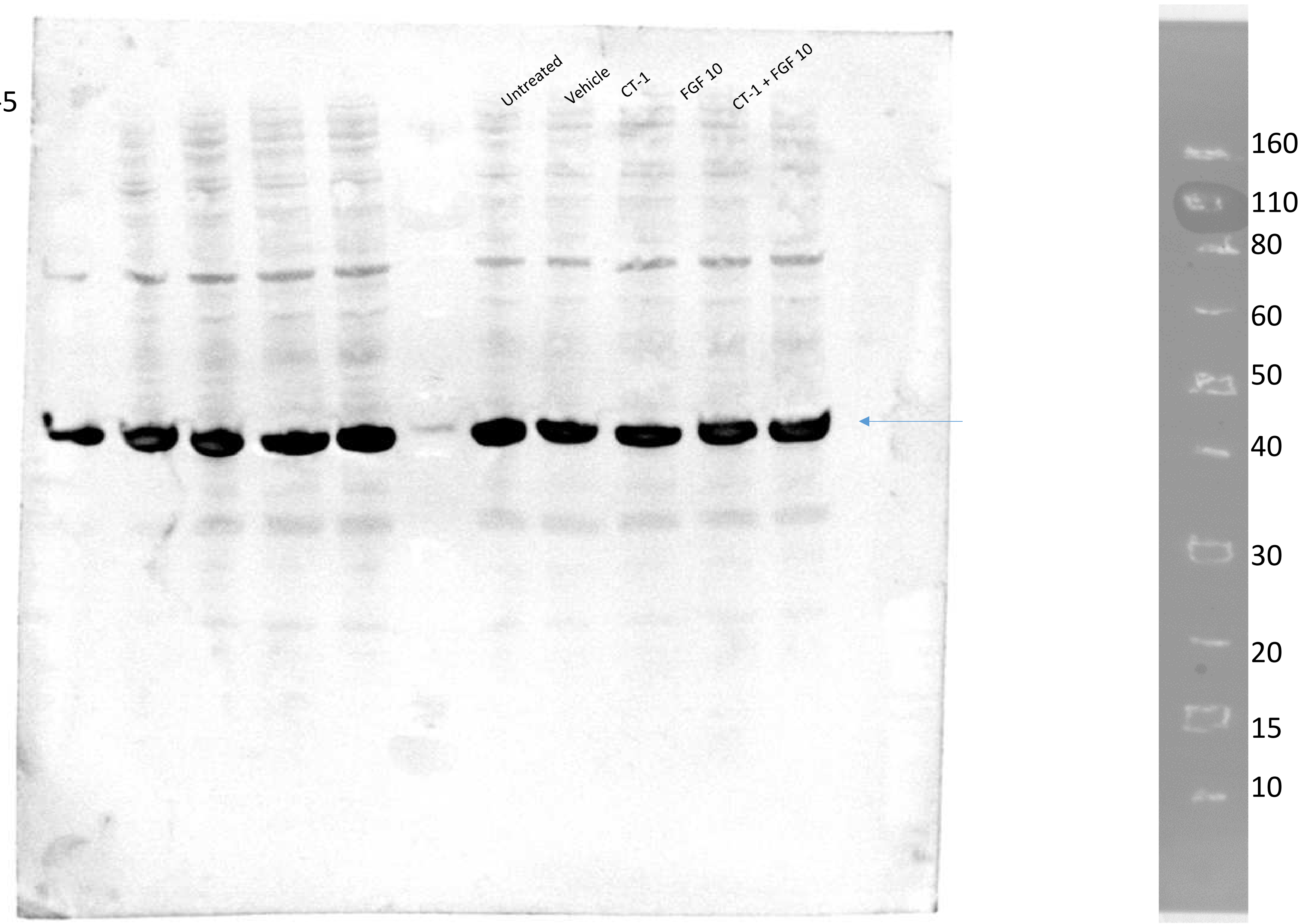

NKX2-5 (34kDa)
N2 and N3

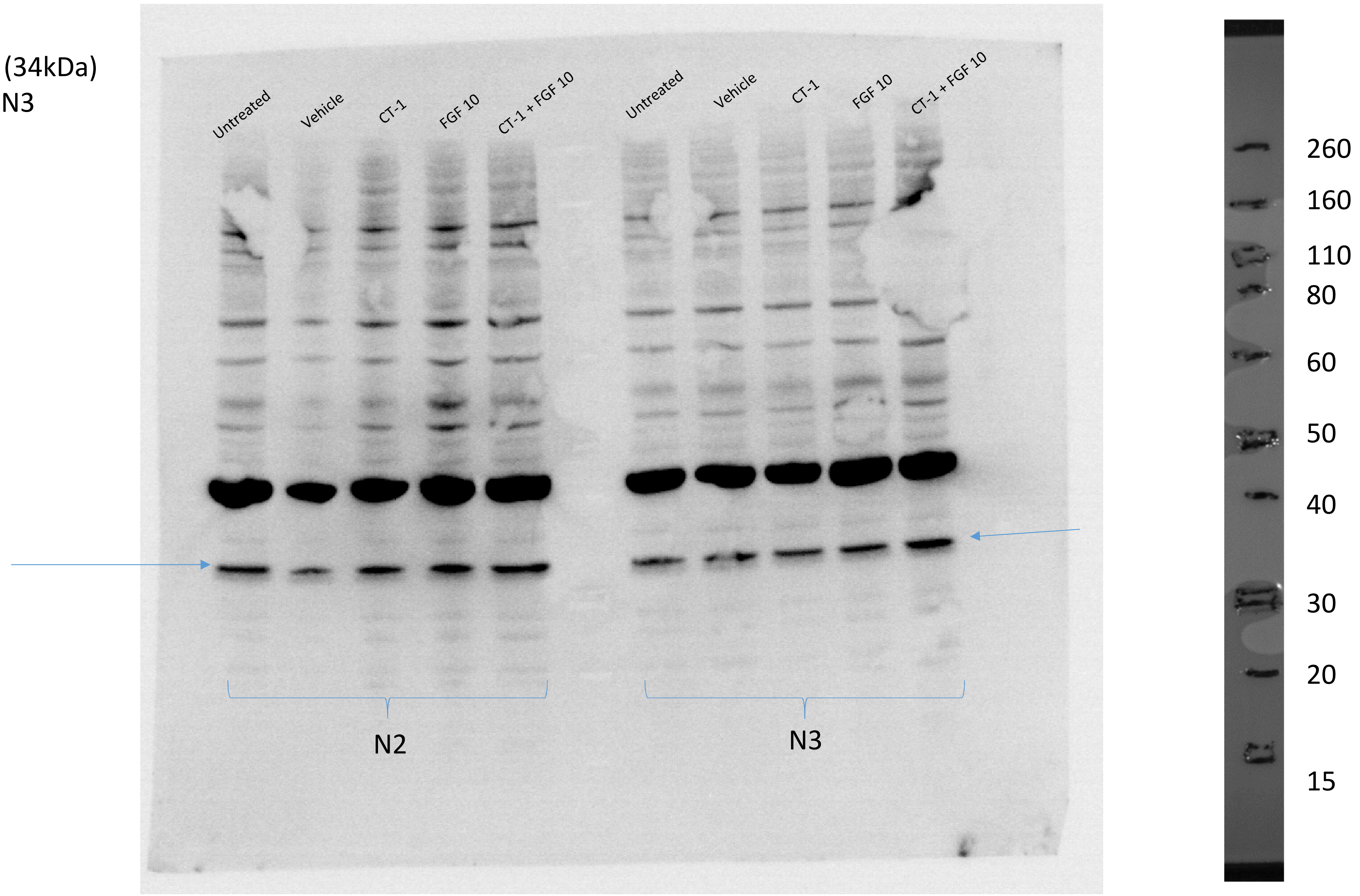

Beta actin (42 kDa)
Housekeeping of NKX2-5
N2 and N3

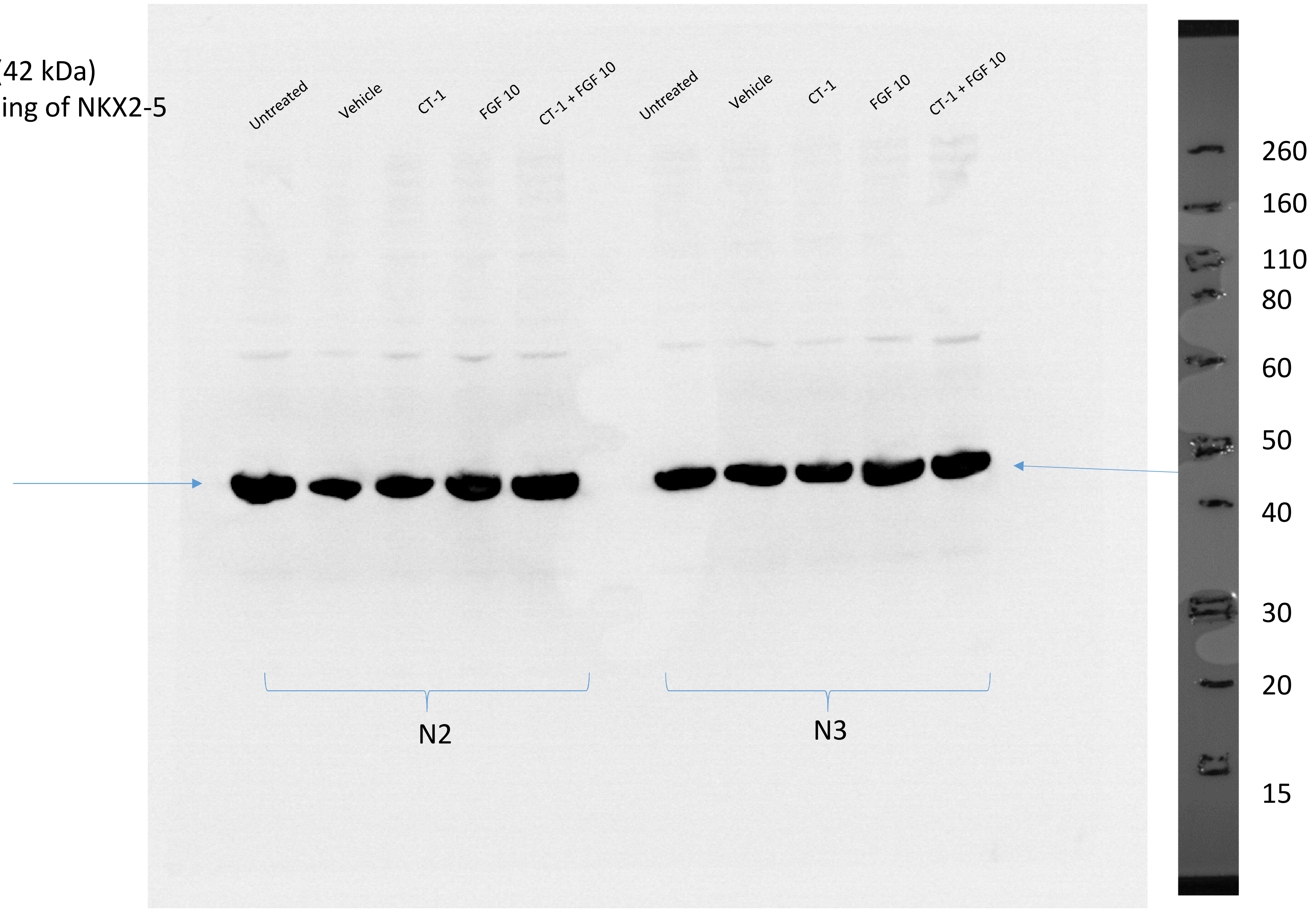

NKX2-5 (34kDa)
N4

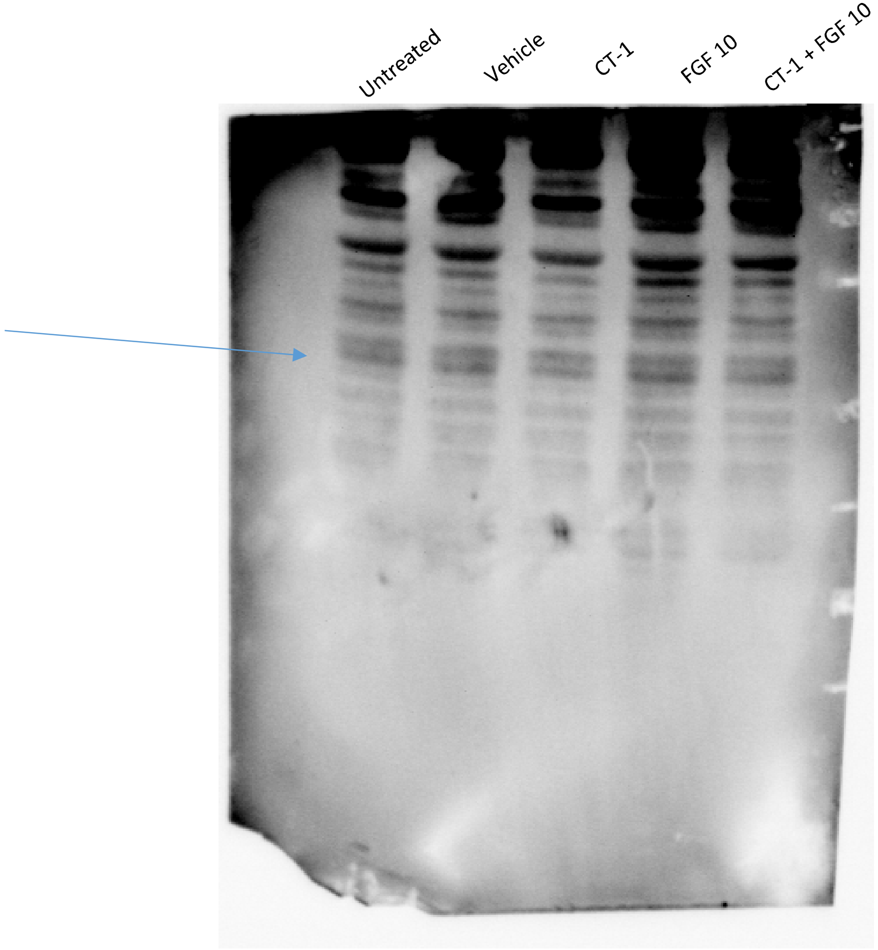

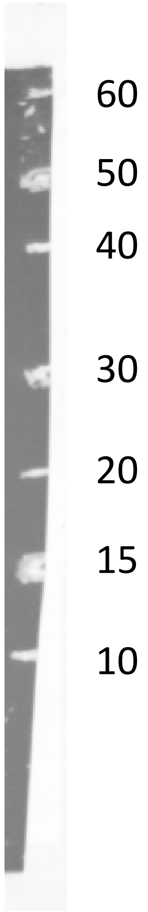

Beta actin (42 kDa)
Housekeeping of NKX2-5
N4

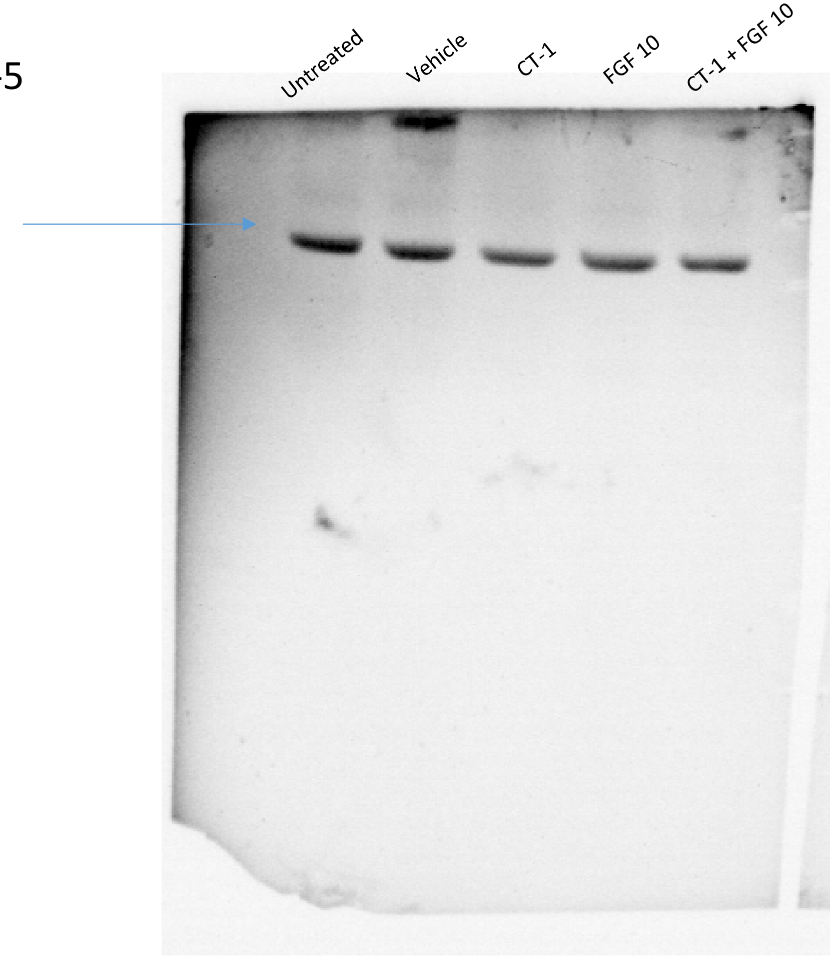

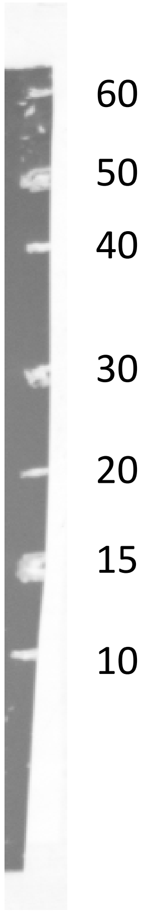

YAP-1 and Housekeeping WB replicates

YAP-1 (70kDa) and vinculin(124 kDa) as Housekeeping (N1 representative and N2)

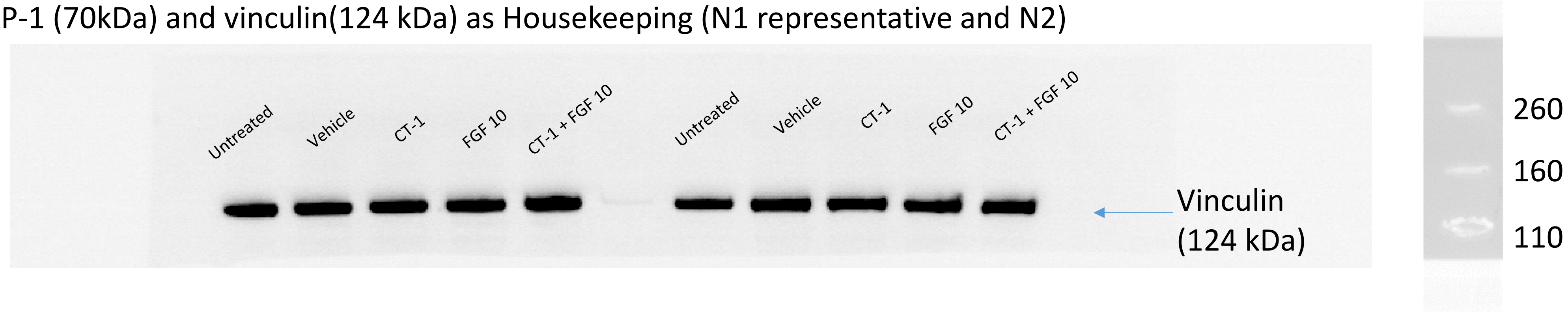

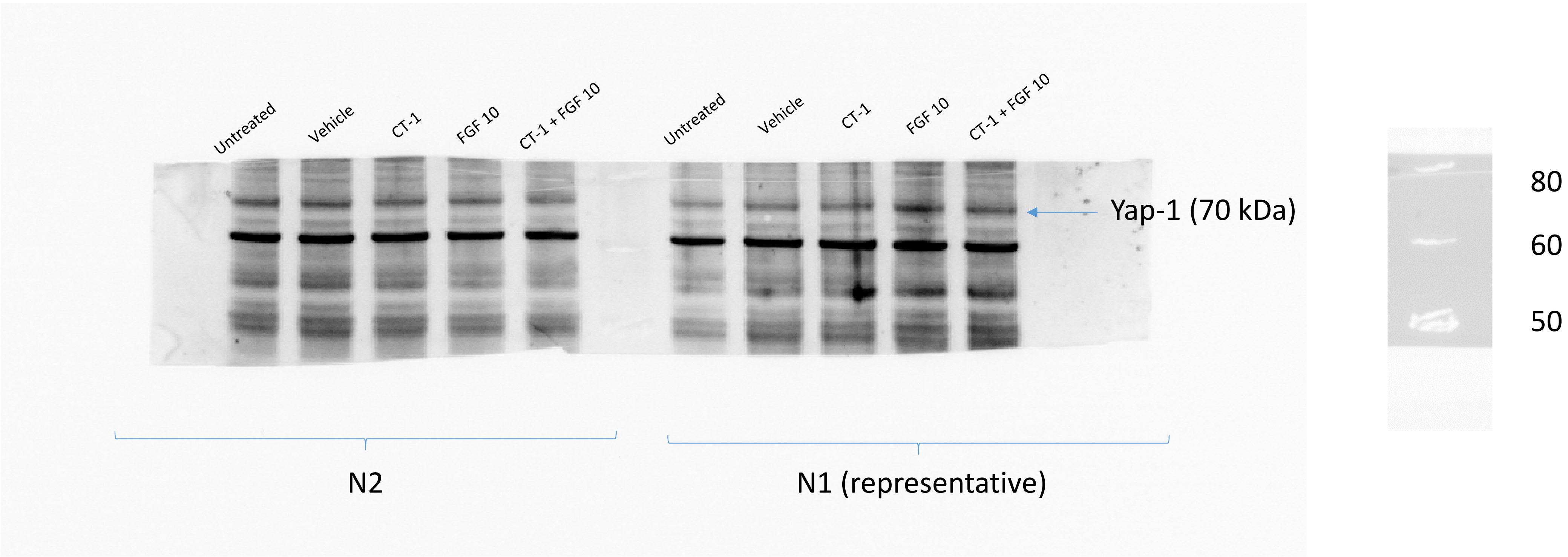

YAP-1 (N3)

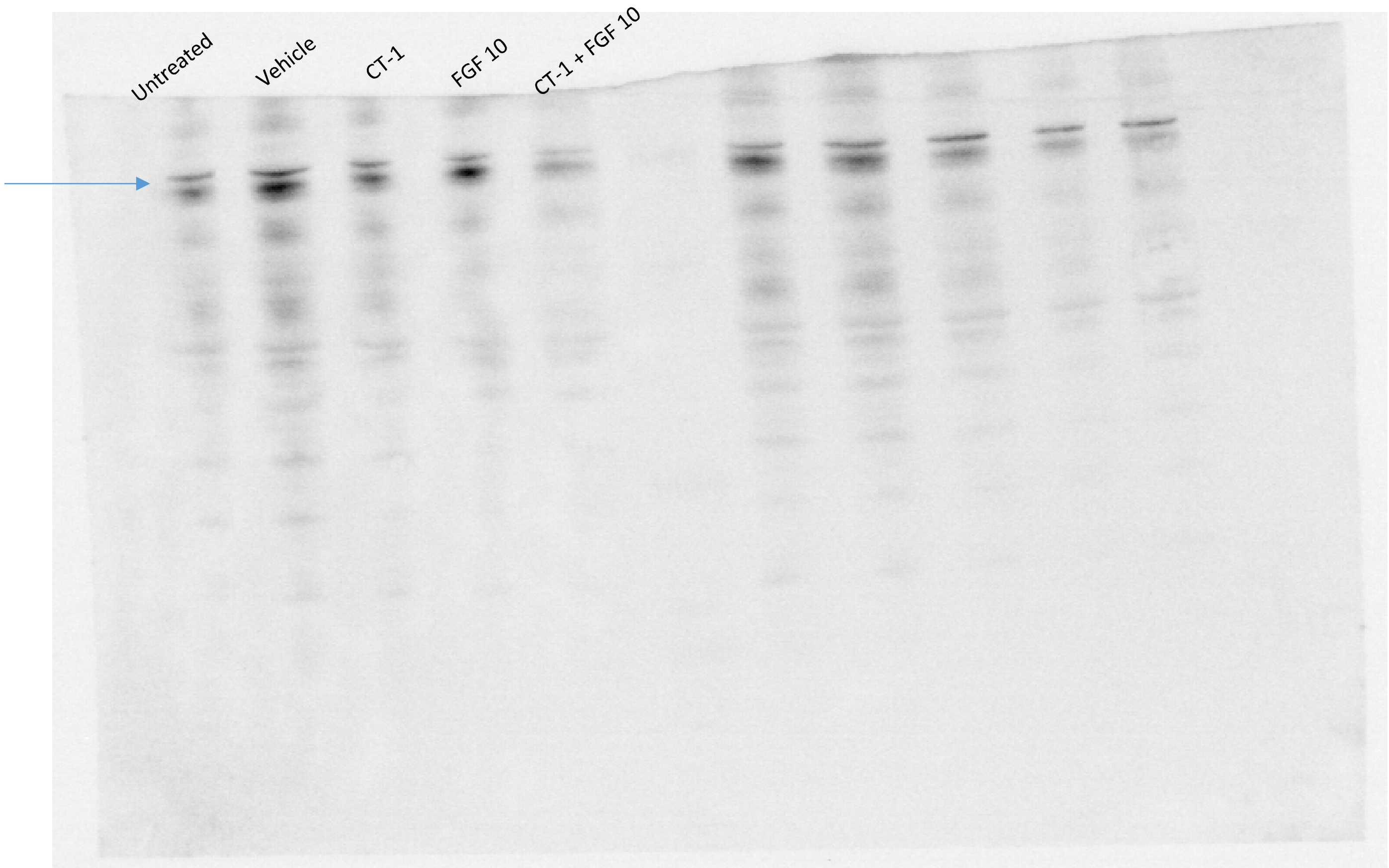

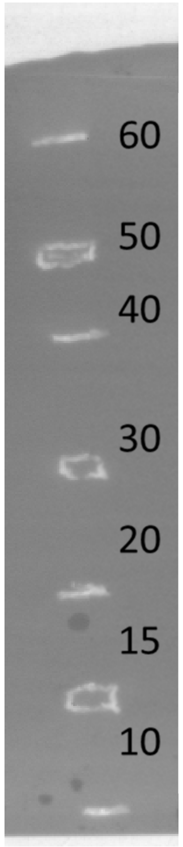

Vinculin as housekeeping of YAP-1 (N3)

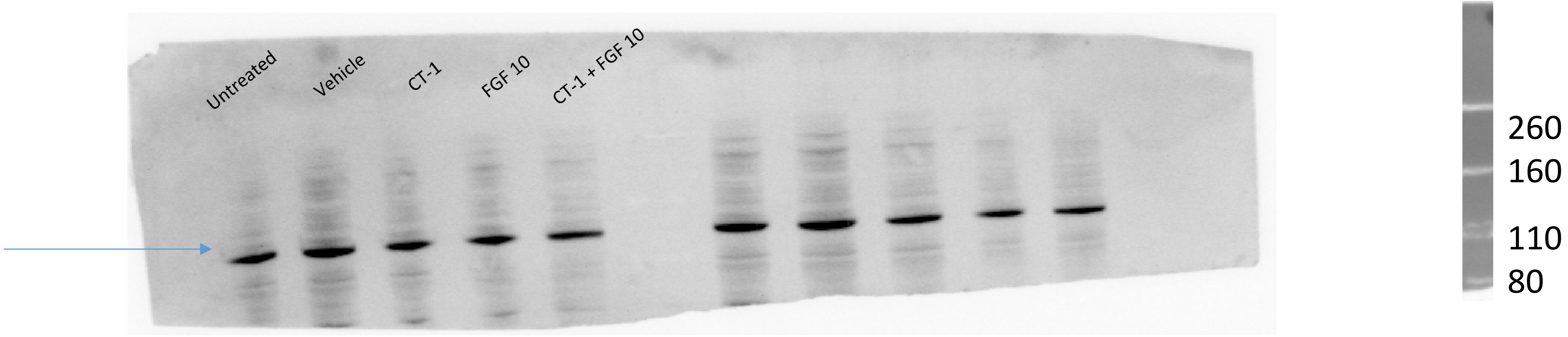

YAP-1 (N4)

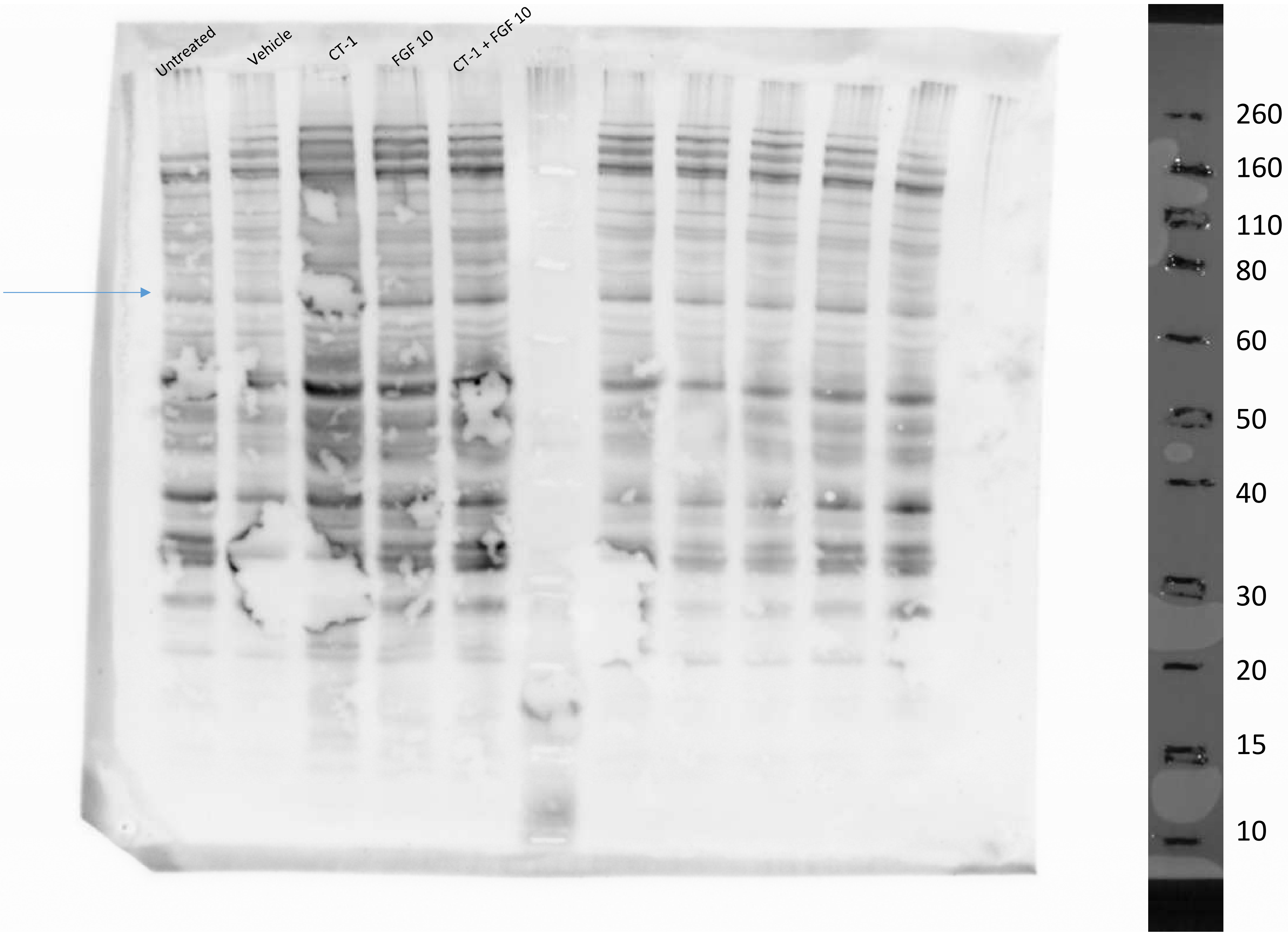

Untreated
Vehicle
CT-1
FGF 10
CT-1 + FGF 10
260
160
110
80
60
50
40
30
20
15
10

Vinculin as housekeeping of YAP-1 (N4)

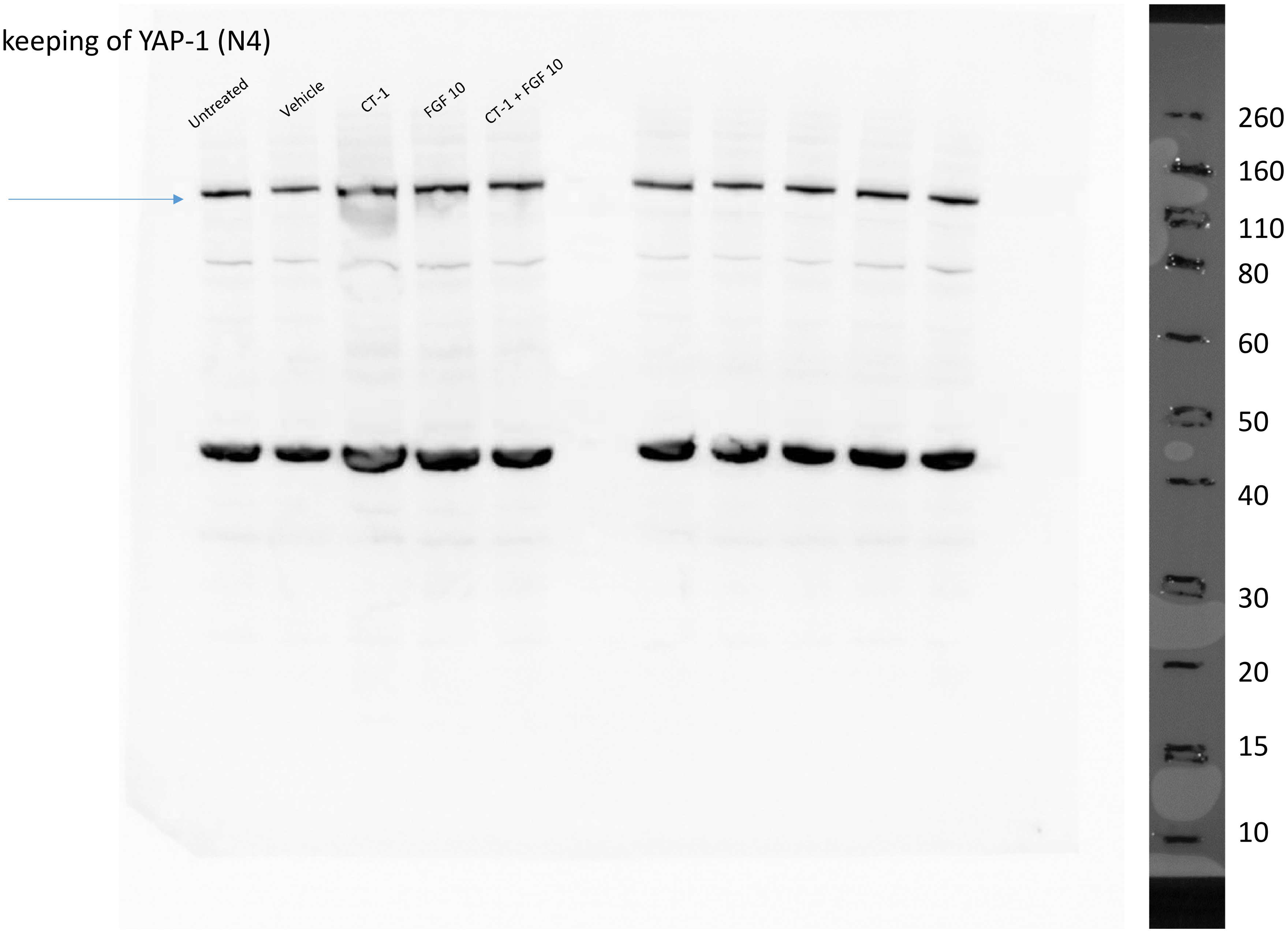

MLC2a and Housekeeping WB replicates

MLC2a (19kDa) and Beta actin (42 kDa) as Housekeeping (representative N1)

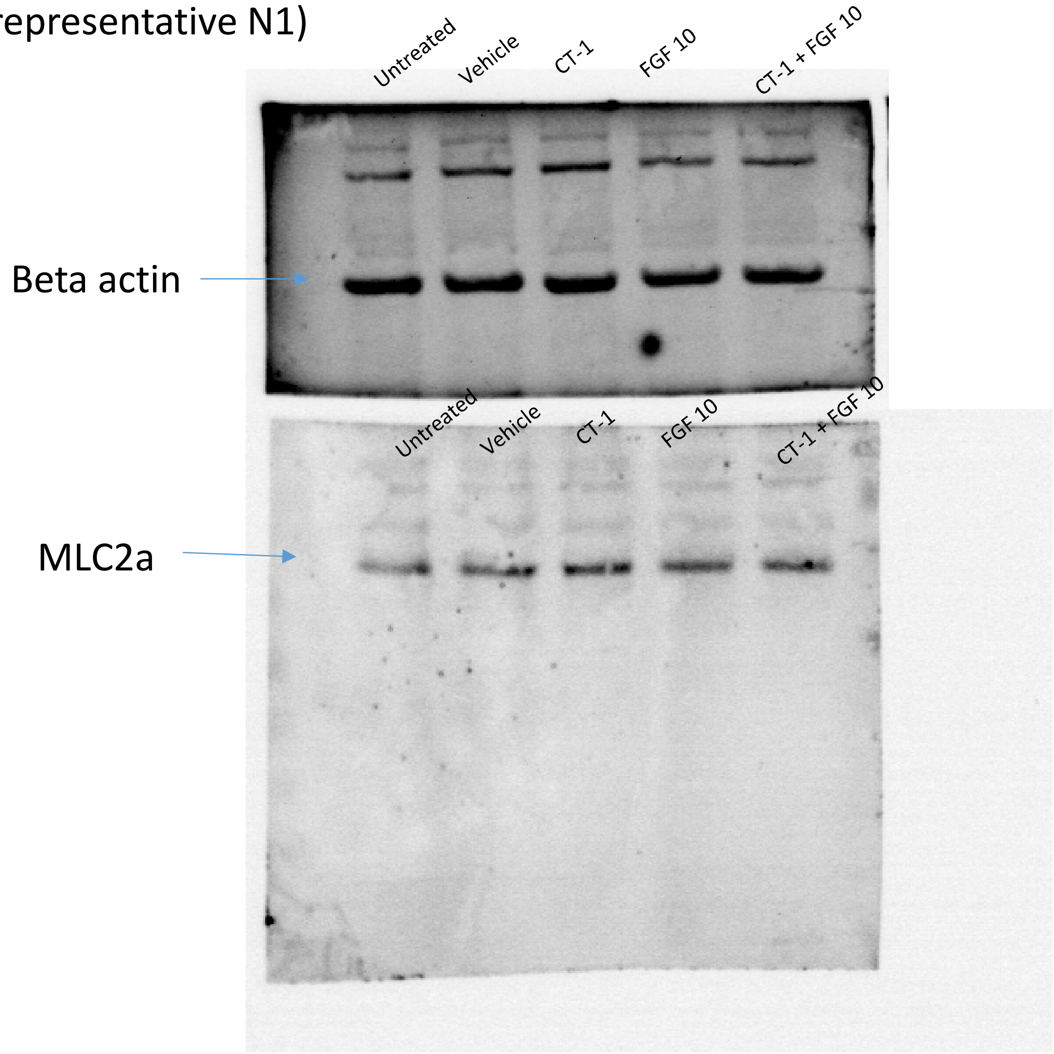

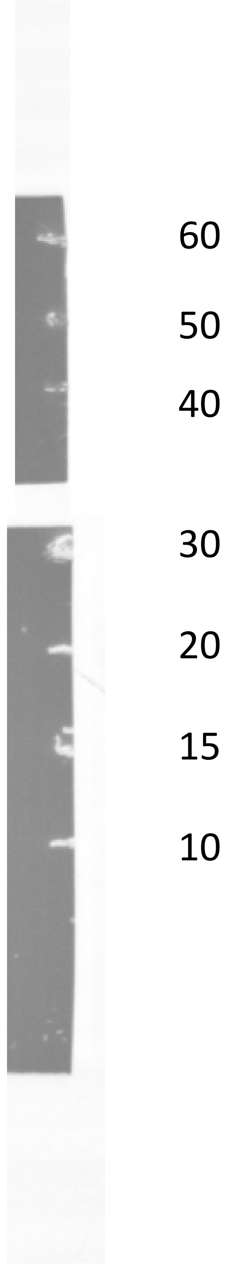

MLC2a (19 kDa), N2

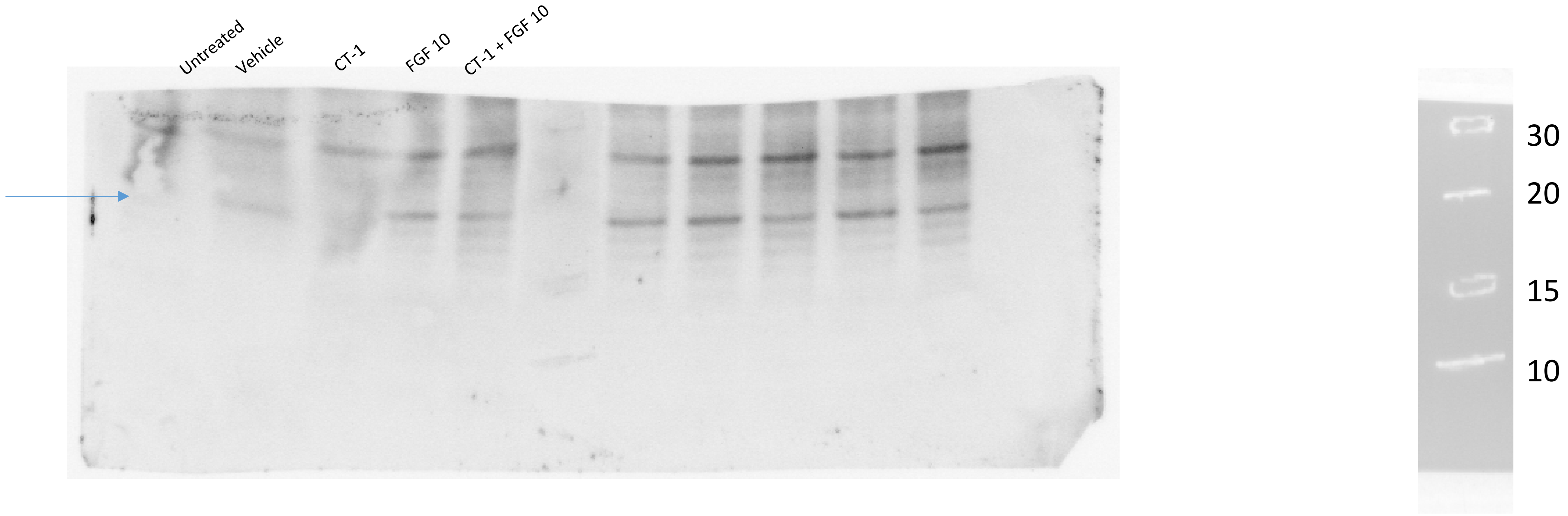

Beta-actin (42 kDa) as housekeeping of MLC-2a (N2)

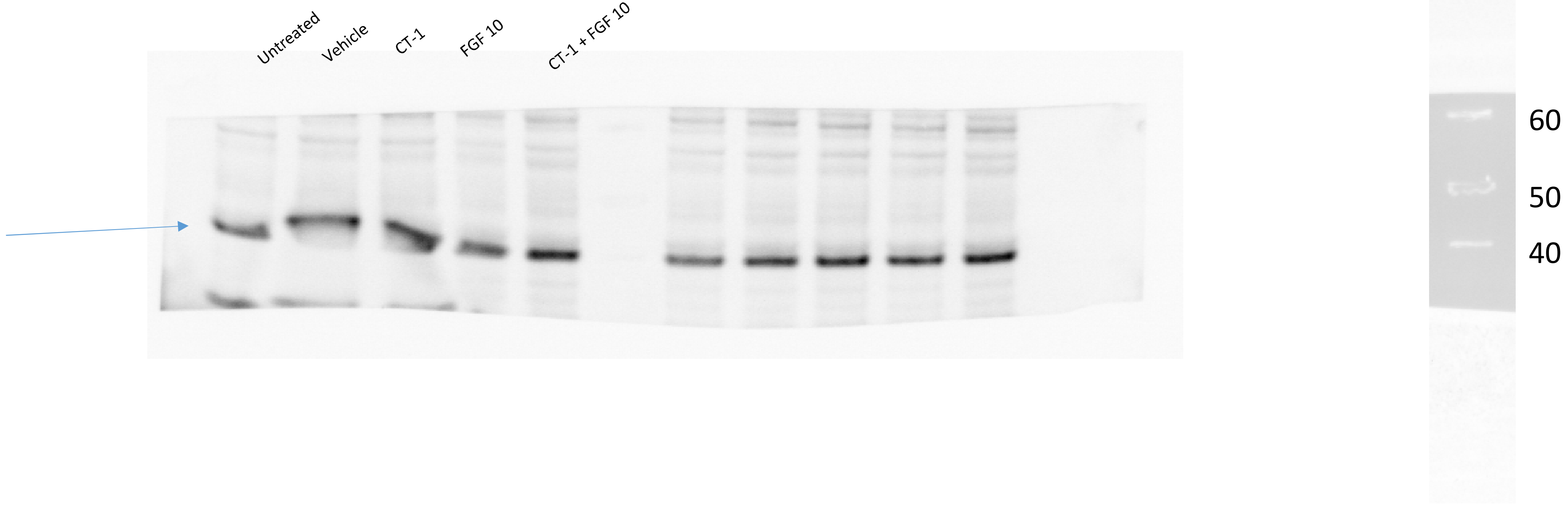

MLC2a (19 kDa), N3

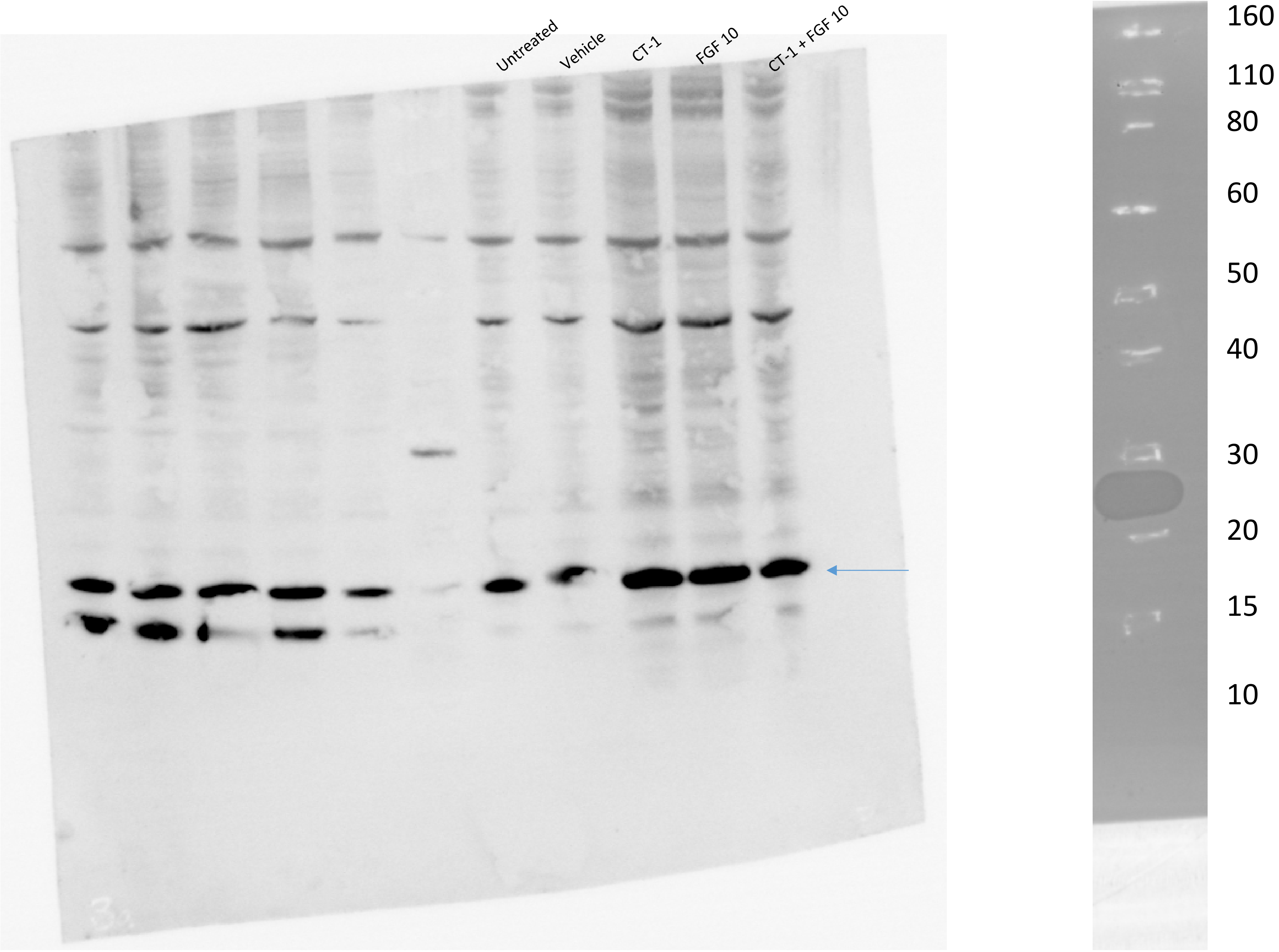

Beta-actin (42 kDa) as housekeeping of MLC-2a (N3)

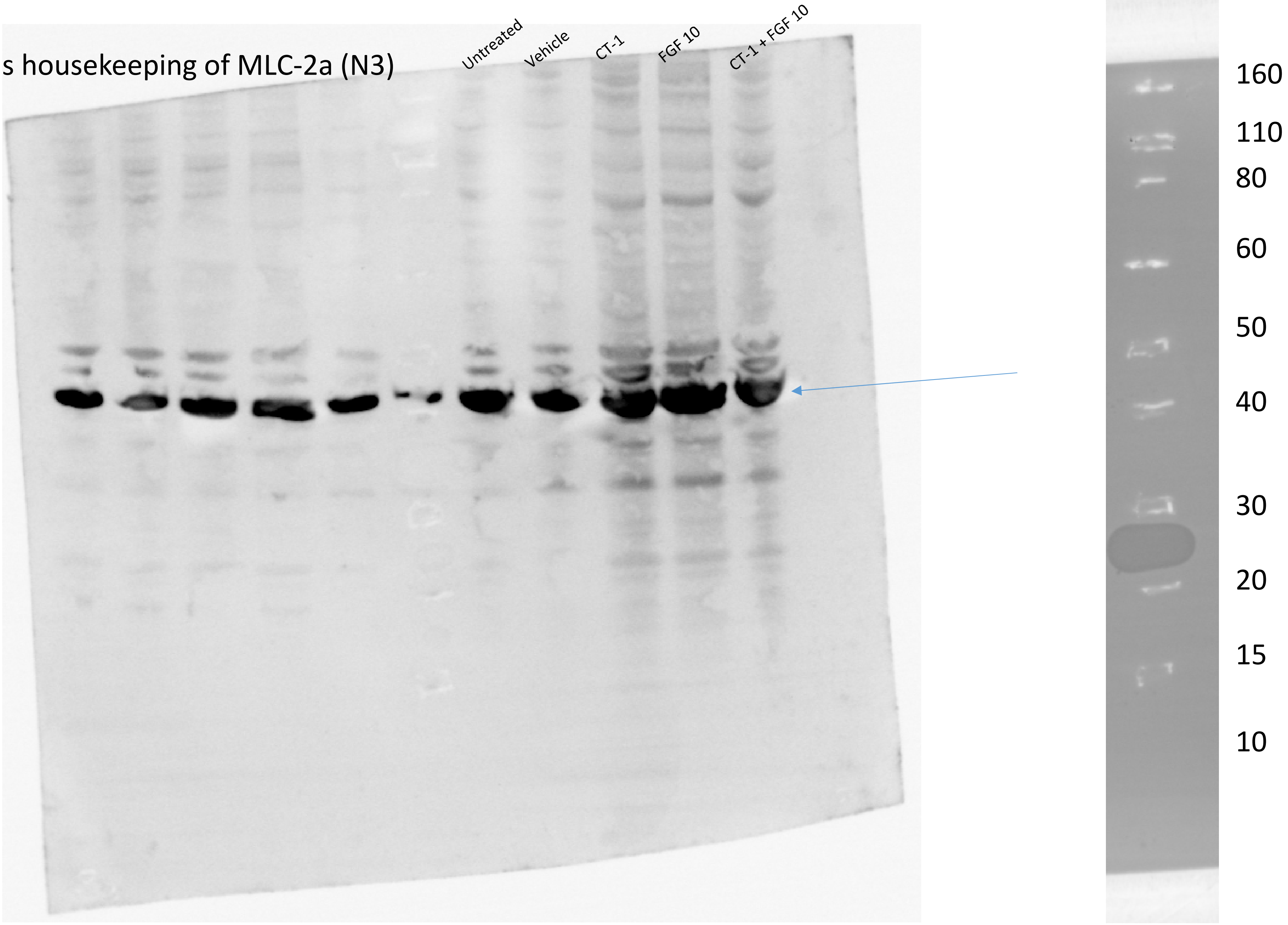

MLC2a (19 kDa), N4

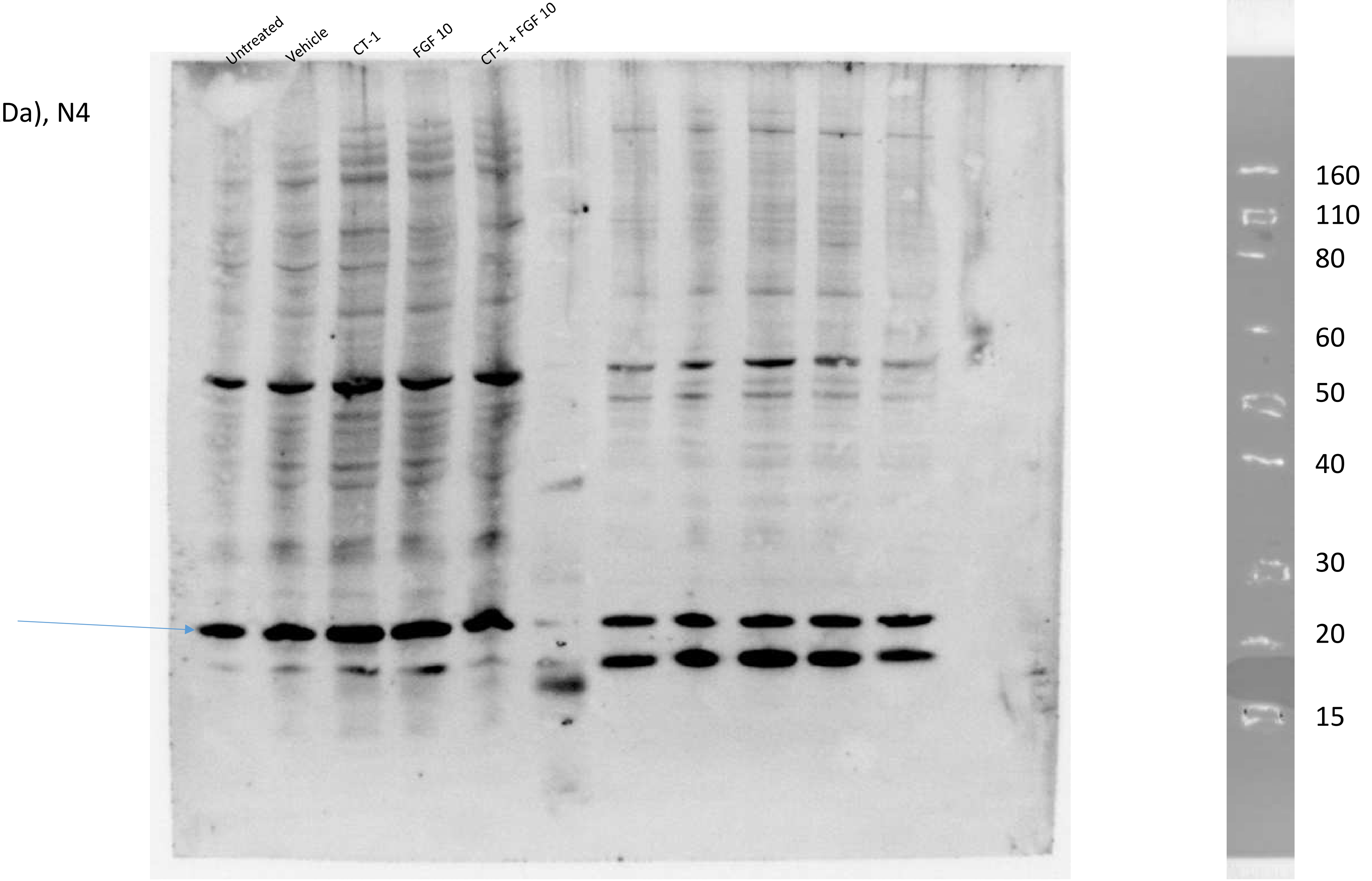

Beta-actin as housekeeping of MLC-2a (N4)

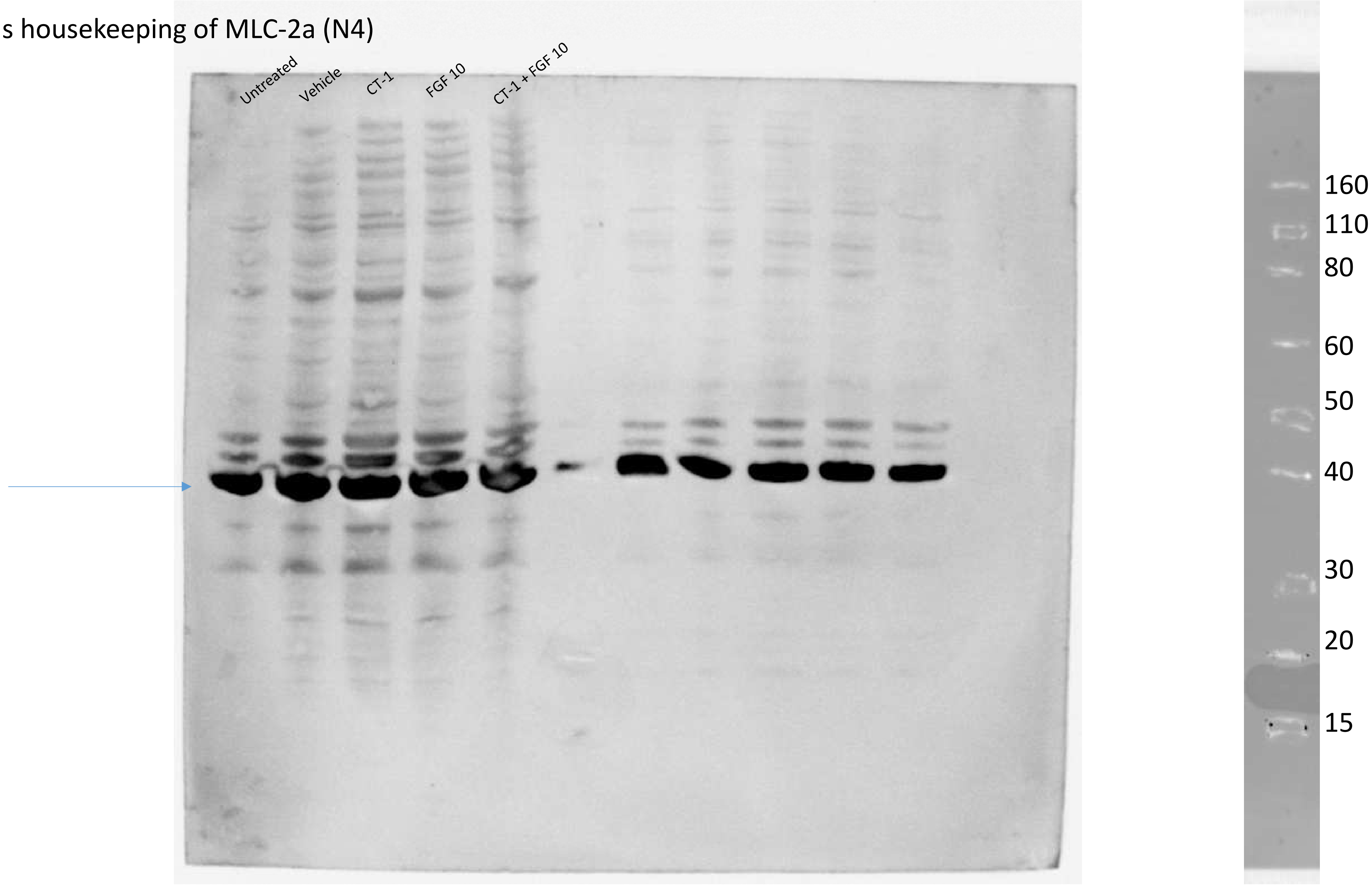

GATA4 and Housekeeping WB replicates

GATA4 (46kDa) (N1 as representative)

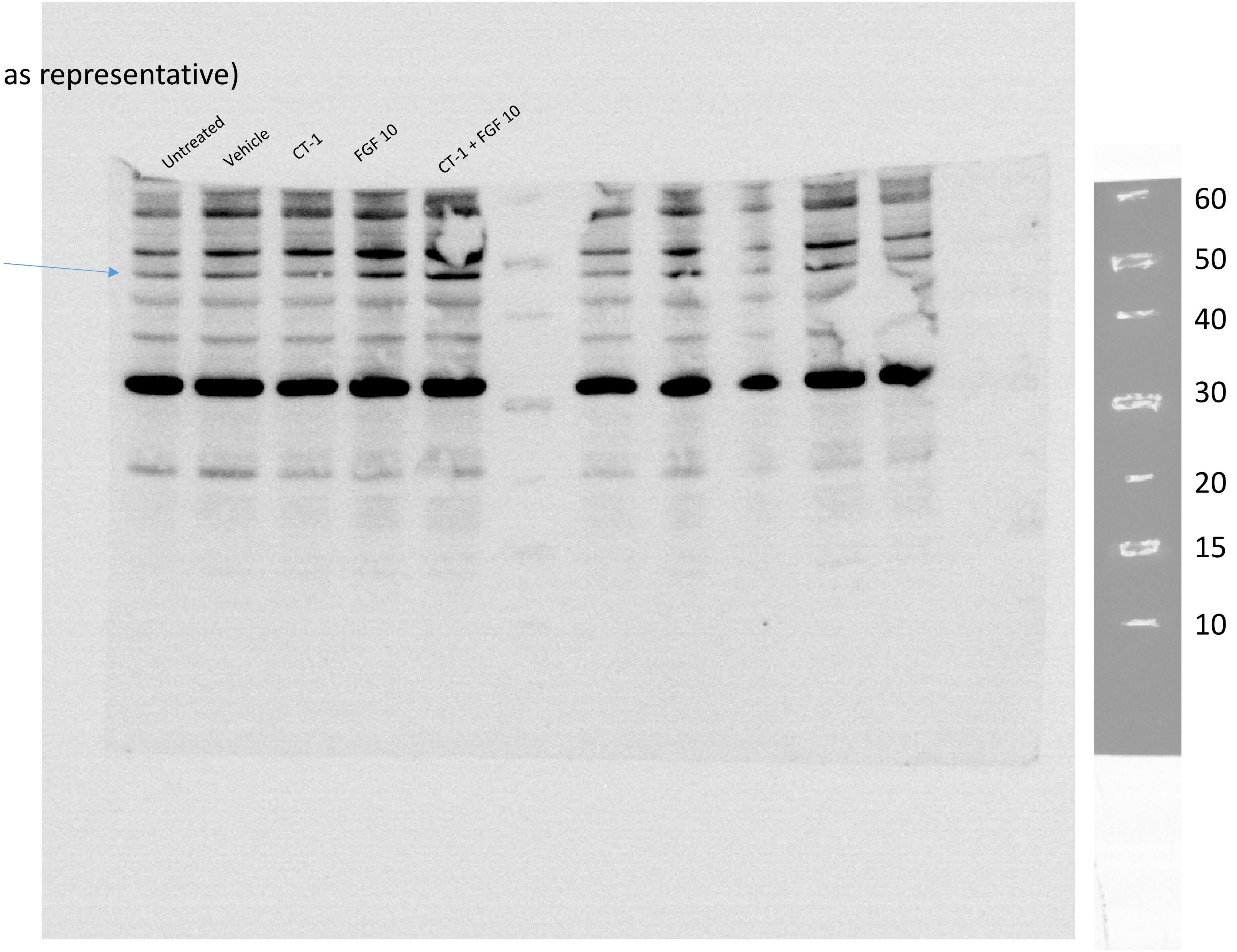

Beta actin (42kDa) (Housekeeping of GATA4), N1

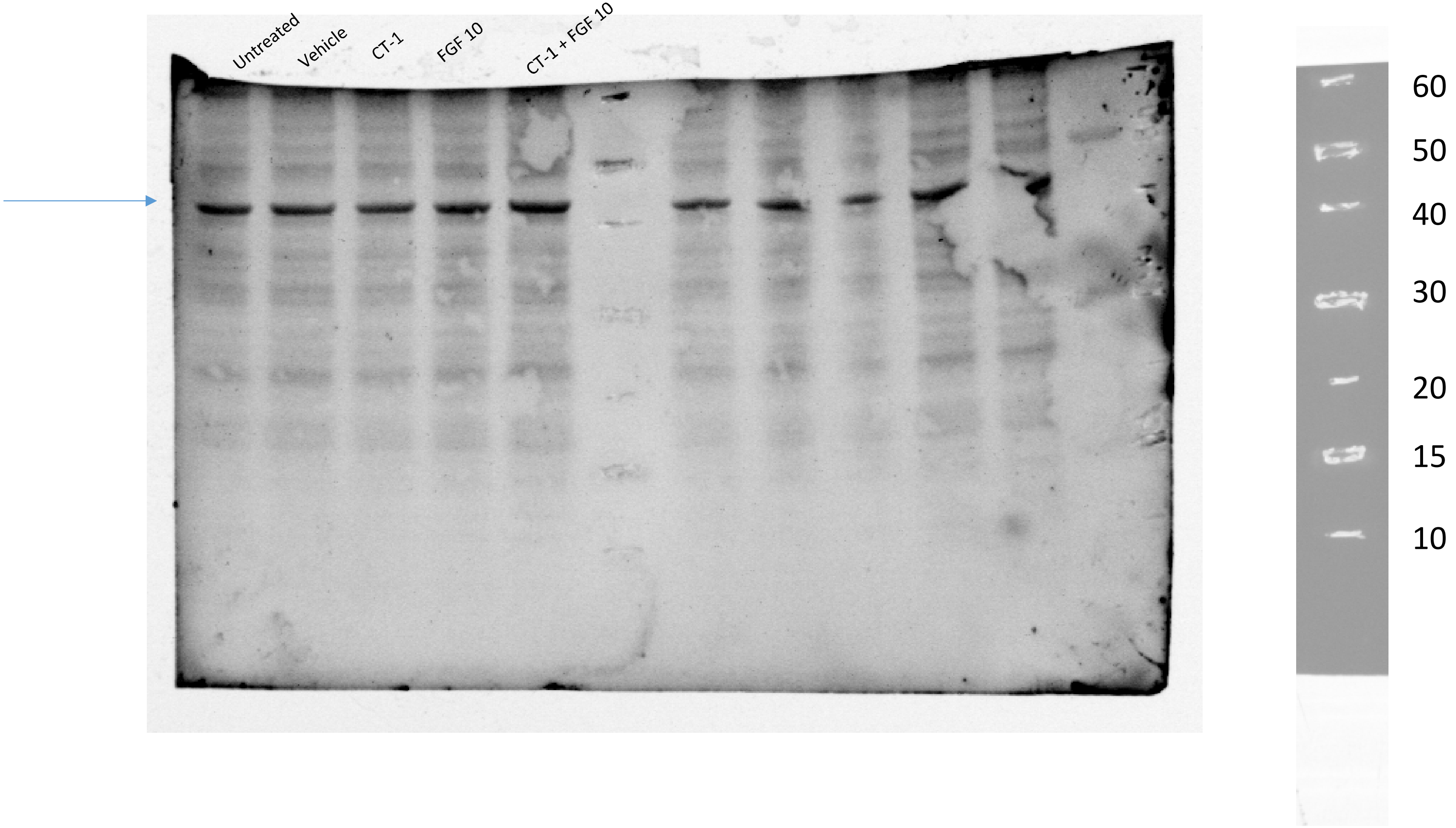

GATA4 46 kDa (N2 and N3)

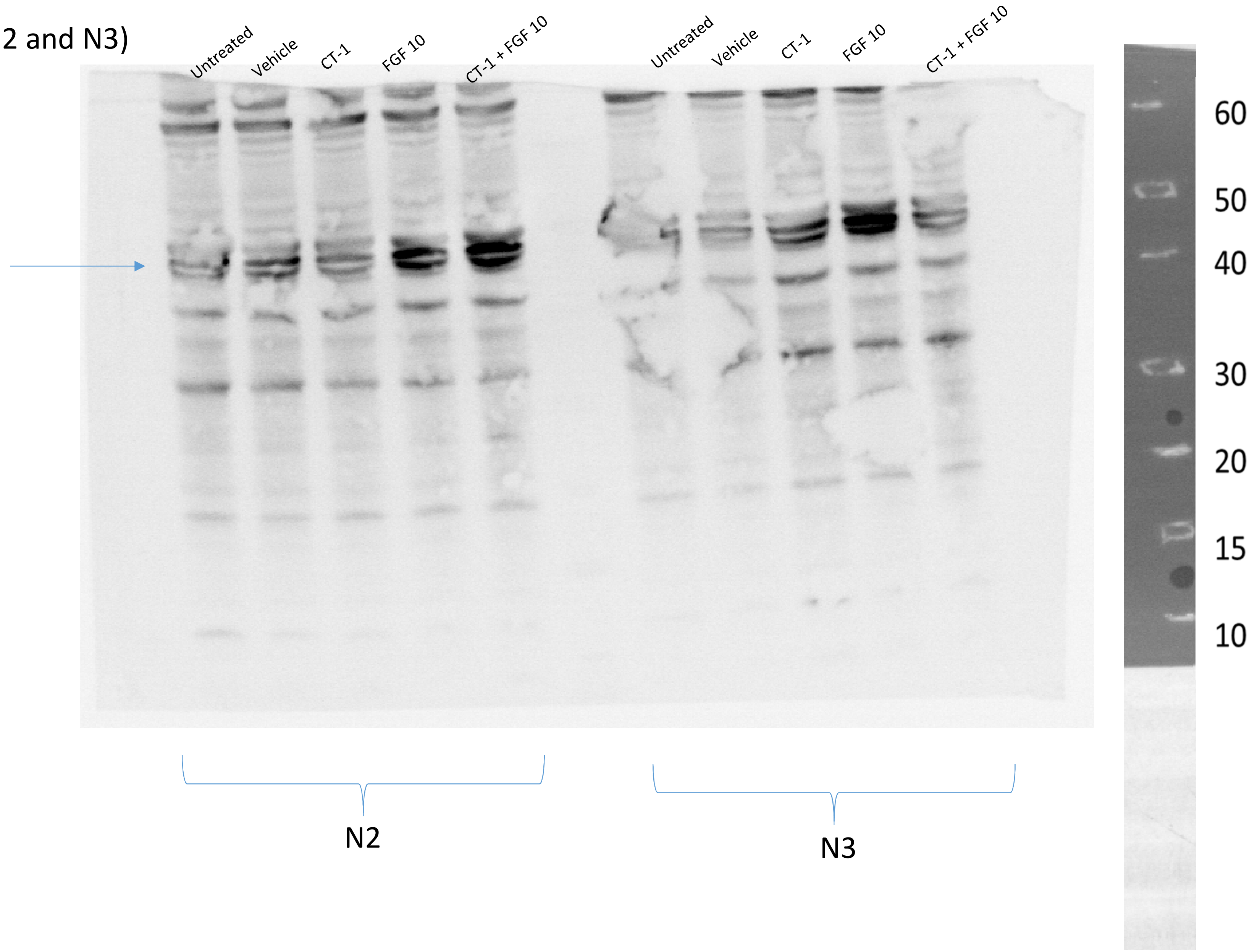

Beta actin (42kDa) (Housekeeping of GATA4), N2 & N3

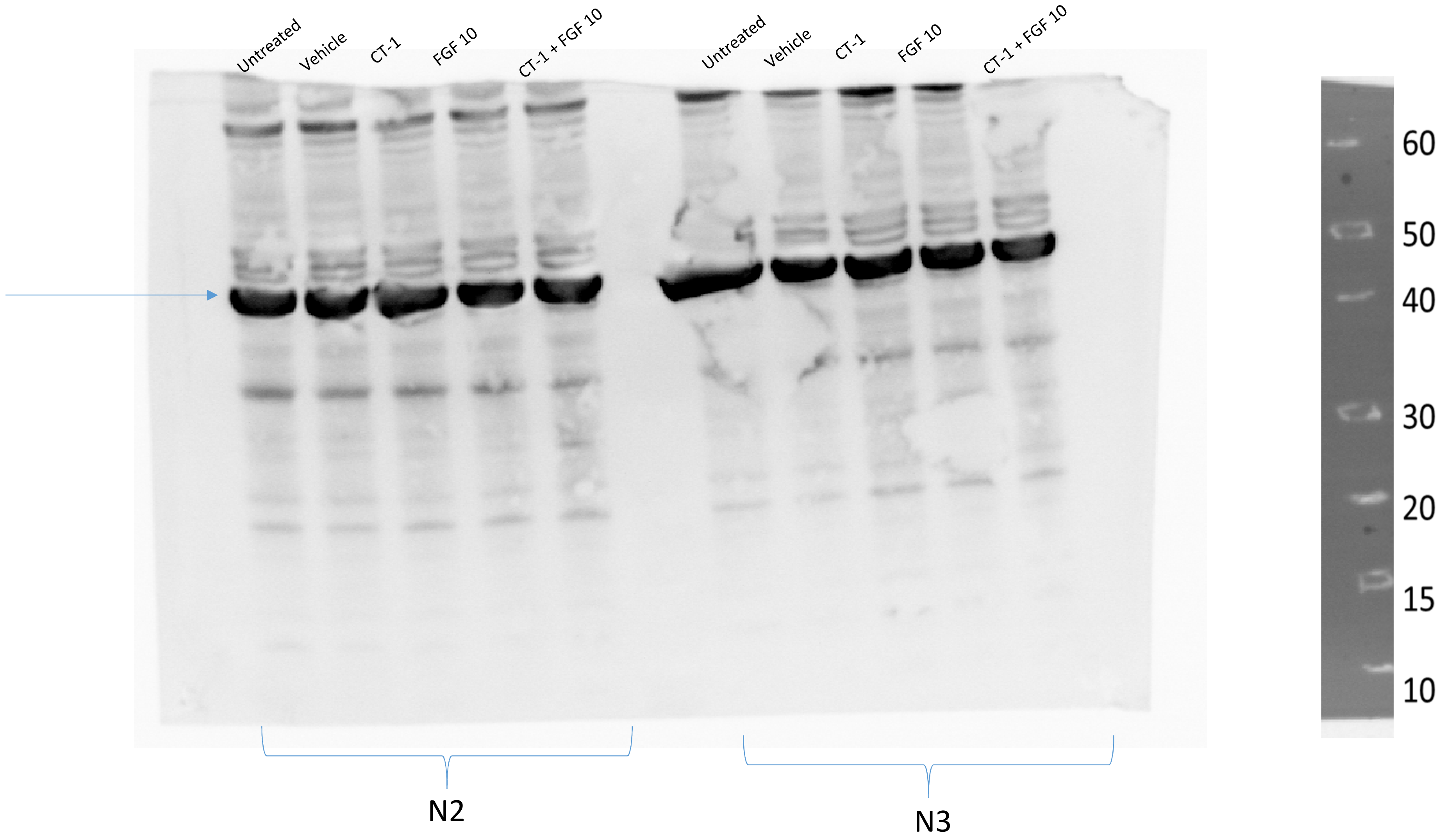

GATA4 46 kDa (N4)

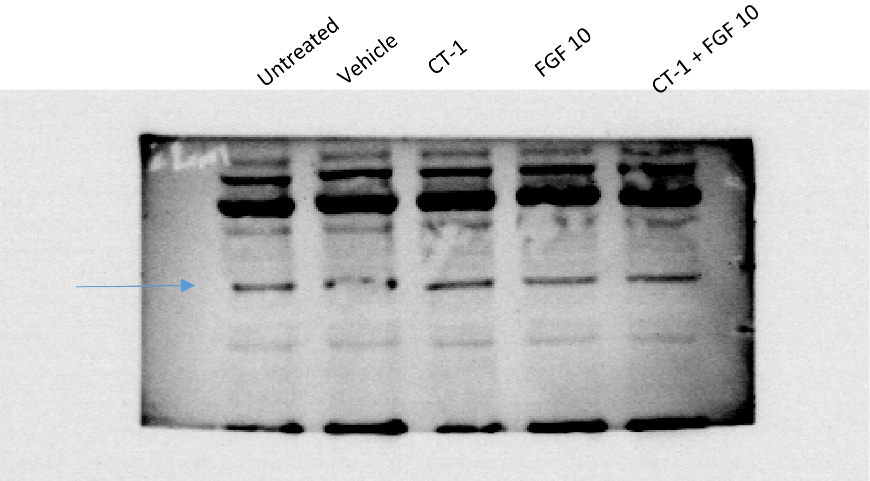

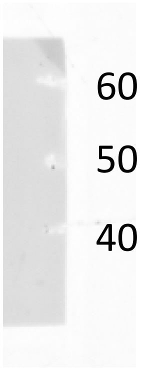

Beta actin (42kDa) (Housekeeping of GATA4), N4:

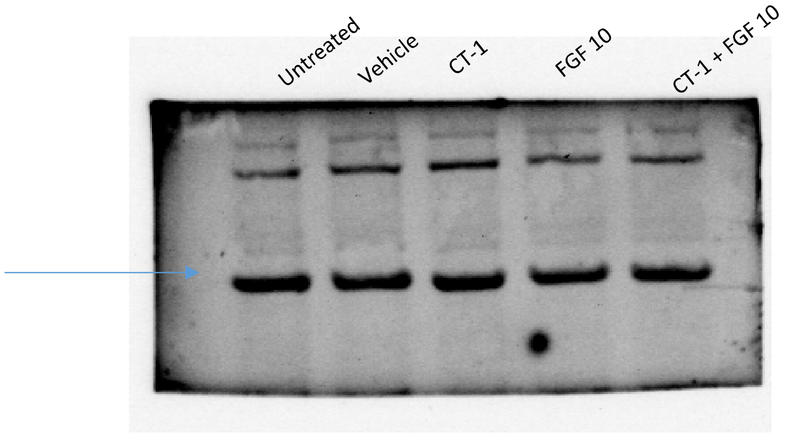

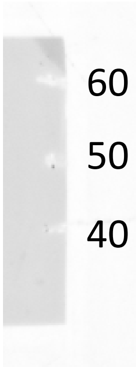

Supplement: Supplementary file 1 [file biology-11-00534-s001.zip › biology-1641253-supplementary.pdf]
